# Disease-first public-data integration with local virtual knockout prioritizes shared proteins linking osteoarthritis and osteoporosis

**DOI:** 10.1371/journal.pone.0359583

**Published:** 2026-10-01

**Authors:** Jiayin Pang, Yu Li, Zhichao Qi, Wenbin Yang

**Affiliations:** 1 The Fifth Clinical College of Guangzhou University of Chinese Medicine, Guangzhou, China; 2 Department of Orthopaedic Surgery, Hirosaki University Graduate School of Medicine, Hirosaki, Japan; 3 Department of Spinal Surgery, The First Affiliated Hospital, Sun Yat-sen University, Guangzhou, China; 4 The Department of Orthopaedics, Guangdong Provincial Second Hospital of Traditional Chinese Medicine, Guangzhou, China; Kyungpook National University School of Medicine, KOREA, REPUBLIC OF

## Abstract

Osteoarthritis and osteoporosis frequently coexist in older adults, but shared molecular programs remain unclear. We developed a disease-first multilayer public-data integration framework to identify shared protein candidates while preserving disease-specific structure before cross-disease comparison. Public bulk transcriptomic datasets from osteoarthritis synovium and cartilage and osteoporosis-related monocytes, femoral bone, and osteogenic stromal compartments were analyzed independently within each disease. Disease-level signatures were generated using differential expression analysis, robust rank aggregation, functional enrichment, and weighted gene co-expression network analysis. A shared axis was defined by concordant dysregulation, shared biological processes, and criteria-positive coexpression-module correspondences, with module matching subsequently calibrated against a size-preserving null model. Candidate genes were mapped to proteins and prioritized by integrating interaction topology, signaling priors, protein annotation, tissue support, disease-association and genetic evidence, local CARNIVAL virtual knockout, and single-cell contextual perturbation analysis. Both diseases showed reproducible signatures enriched in antigen presentation, cytokine regulation, extracellular matrix organization, osteoclast differentiation, ossification, and bone remodeling. Cross-disease comparison yielded 234 criteria-positive module pairs; 53 retained pair-specific support at empirical FDR < 0.05, whereas the total number of criteria-positive pairs did not exceed the global null expectation. Protein-level integration prioritized HLA-DRB1, HSP90AA1, CTSK, RPL7, PRG4, HLA-DRA, CLEC3B, TIMP1, SPP1, and APOE. Local virtual knockout assigned the highest model-derived CARNIVAL scores to HLA-DRB1 and HSP90AA1. Across the evaluated network configurations, HLA-DRB1 exceeded the prespecified score threshold in all four evaluable configurations, whereas HSP90AA1 exceeded it in four of six, indicating greater configuration stability for HLA-DRB1. Primary matched-null calibration of 39 evaluable candidate-compartment pairs retained nine pairs at global FDR < 0.05. External quality-control sensitivity analysis retained 125,090 of 161,470 cells and identified 10 globally FDR-supported pairs. Although only two of the nine primary pairs remained supported in the same compartment, five of the six primary supported genes retained evidence in at least one compartment. Recalculation using the quality-controlled cell-context evidence retained all primary top 10 proteins, with HLA-DRB1 and HSP90AA1 remaining the two highest-ranked candidates. Composition-aware bulk sensitivity analysis retained the direction of 36 of 40 candidate–cohort effects, while matched transcriptional-program adjustment retained 24 of 27 effects and showed greater attenuation of MHC-II candidates than of ribosomal or protein-folding candidates. These findings support a shared osteoimmune–matrix-remodeling protein axis linking osteoarthritis and osteoporosis and provide candidates for future experimental validation.

## Introduction

Osteoarthritis (OA) and osteoporosis (OP) are among the most common musculoskeletal disorders in ageing societies. Clinically, OA and OP frequently coexist in the same individual. Traditionally, OA has been regarded as a disease of articular cartilage wear or degeneration, whereas OP has been viewed as a systemic skeletal metabolic disorder characterized by reduced bone mass and increased bone fragility. Earlier studies on the relationship between OA and OP proposed an inverse association, suggesting that patients with OA may have higher bone mineral density and a lower risk of OP [1]. However, recent reviews indicate that the relationship between these two conditions is not simply antagonistic, but is influenced by age, sex, body mass index (BMI), skeletal site of measurement, disease stage, bone quality, mechanical loading, and inflammatory status [2].

High-quality evidence in recent years has established that OA is not merely a disease of cartilage wear, but rather a heterogeneous “whole-joint disease” involving cartilage, synovium, subchondral bone, infrapatellar fat pad, meniscus, ligaments, and periarticular muscles. Its onset and progression are driven by multiple factors, including mechanical stress, metabolic abnormalities, low-grade inflammation, cellular senescence, oxidative stress, impaired autophagy, and pain-related neuroregulation [3–6]. The central pathological feature of OP is an imbalance in bone remodeling, in which osteoclast-mediated bone resorption exceeds osteoblast-mediated bone formation. Ageing, estrogen deficiency, chronic inflammation, oxidative stress, immune dysregulation, and altered lineage commitment of bone marrow mesenchymal stem cells all contribute to the development of OP. Particularly in older individuals, the immune system and bone remodeling system are closely interconnected through signaling pathways involving RANKL/RANK/OPG, TNF-α, IL-1β, IL-6, NF-κB, and NFATc1, collectively forming the pathological basis of osteoimmunology [7–9]. Therefore, although OA and OP differ in their tissue-level phenotypes, they may share overlapping mechanisms related to cellular stress, inflammation, senescence, autophagy, and dysregulated tissue remodeling. OA complicated by OP may represent a distinct osteoarticular comorbidity phenotype, in which the underlying mechanisms are not a simple summation of OA and OP, but rather the convergence of stress responses, inflammation, and abnormal bone–joint remodeling in the context of ageing.

Despite this emerging concept, defining the shared biology linking OA and OP remains challenging. OA studies are commonly based on synovium or cartilage, whereas OP studies often involve circulating monocytes, femoral bone, or osteogenic stromal compartments. In the present study, we sought to address this problem by developing a disease-first, multilayer public-data integration framework to investigate shared OA-OP pathology. Rather than merging heterogeneous datasets at the outset, we first characterized robust disease signatures within OA and within OP, and then used multilayer evidence to identify a shared pathological axis linking the two conditions. On this basis, we further prioritized candidate proteins with support from network context, disease relevance, and cellular localization. The aim of the study was to derive a biologically interpretable set of shared OA-OP protein candidates that may be useful for future mechanistic and translational investigation.

## Results

### Disease-first cohort organization preserved within-disease structure before cross-disease inference

The study design and included datasets are summarized in Fig 1A–D and Table 1. The OA bulk layer included GSE55235, GSE55457 [10], GSE82107 [11], and GSE117999 [12], spanning synovium and cartilage. The OP bulk layer included GSE56814, GSE56815 [13], GSE35958 [14], and GSE230665 [15], spanning circulating monocytes, femoral bone, and osteogenic stromal compartments. The single-cell layer included GSE216651 [16] and GSE152805 [17] for OA-related context and GSE147287 [18] for OP-related context, with CELLxGENE [19] used as an auxiliary reference for cell-type interpretation.

**Table 1:** Public datasets included in the study. Summary of bulk and single-cell public datasets used in the disease-first workflow, including their analytical role in the manuscript.

Dataset	Disease	Layer	Material / focus used in study	Platform	Sample summary	Use in manuscript
GSE55235	OA	bulk	synovium	GPL96	10 OA / 10 healthy	Bulk differential analysis and OA RRA
GSE55457	OA	bulk	synovium	GPL96	10 OA / 10 healthy	Bulk differential analysis and OA RRA
GSE82107	OA	bulk	synovium	GPL570	10 OA / 7 healthy	Bulk differential analysis and OA RRA
GSE117999	OA	bulk	cartilage	Illumina array	12 OA / 12 healthy	Bulk differential analysis and OA RRA
GSE56814	OP	bulk	circulating monocytes	GPL5175	31 low BMD / 42 high BMD	Bulk differential analysis and OP RRA
GSE56815	OP	bulk	circulating monocytes	GPL96	40 low BMD / 40 high BMD	Bulk differential analysis and OP RRA
GSE35958	OP	bulk	osteogenic mesenchymal stromal cells	Array	5 OP / 4 control	Bulk differential analysis and primary OP RRA; not eligible for WGCNA because n = 9.
GSE230665	OP	bulk	femoral bone	GPL10332	12 OP / 3 control	Bulk differential analysis and OP RRA
GSE216651	OA	single-cell	IPFP synovium; OA fibroblast/macrophage perturbation	GPL24676	5 OA synovium / 4 healthy synovium donors	Primary OA single-cell case-control cohort and external quality-control sensitivity analysis
GSE152805	OA	single-cell	paired OA synovium-cartilage atlas; tissue-state support only	GPL20301	paired OA synovium-cartilage atlas; no healthy/control comparator	Descriptive OA atlas support and external quality-control cell-retention assessment (not used for case-control perturbation)
GSE147287	OP	single-cell	bone marrow; BM-MSC and osteoclast-precursor perturbation	GPL24676	1 OP marrow group / 1 OA marrow comparator group	Contextual OP marrow analysis and external quality-control sensitivity analysis for BM-MSCs and osteoclast precursors
CELLXGENE CENSUS	Reference	single-cell	bone marrow/synovium reference atlas for annotation	Census	reference atlas	Reference for annotation transfer and baseline context

All bulk cohorts contributed to cohort-level differential analysis and disease-specific RRA; WGCNA was performed only within eligible tissue- and platform-consistent blocks.

**Fig 1:**
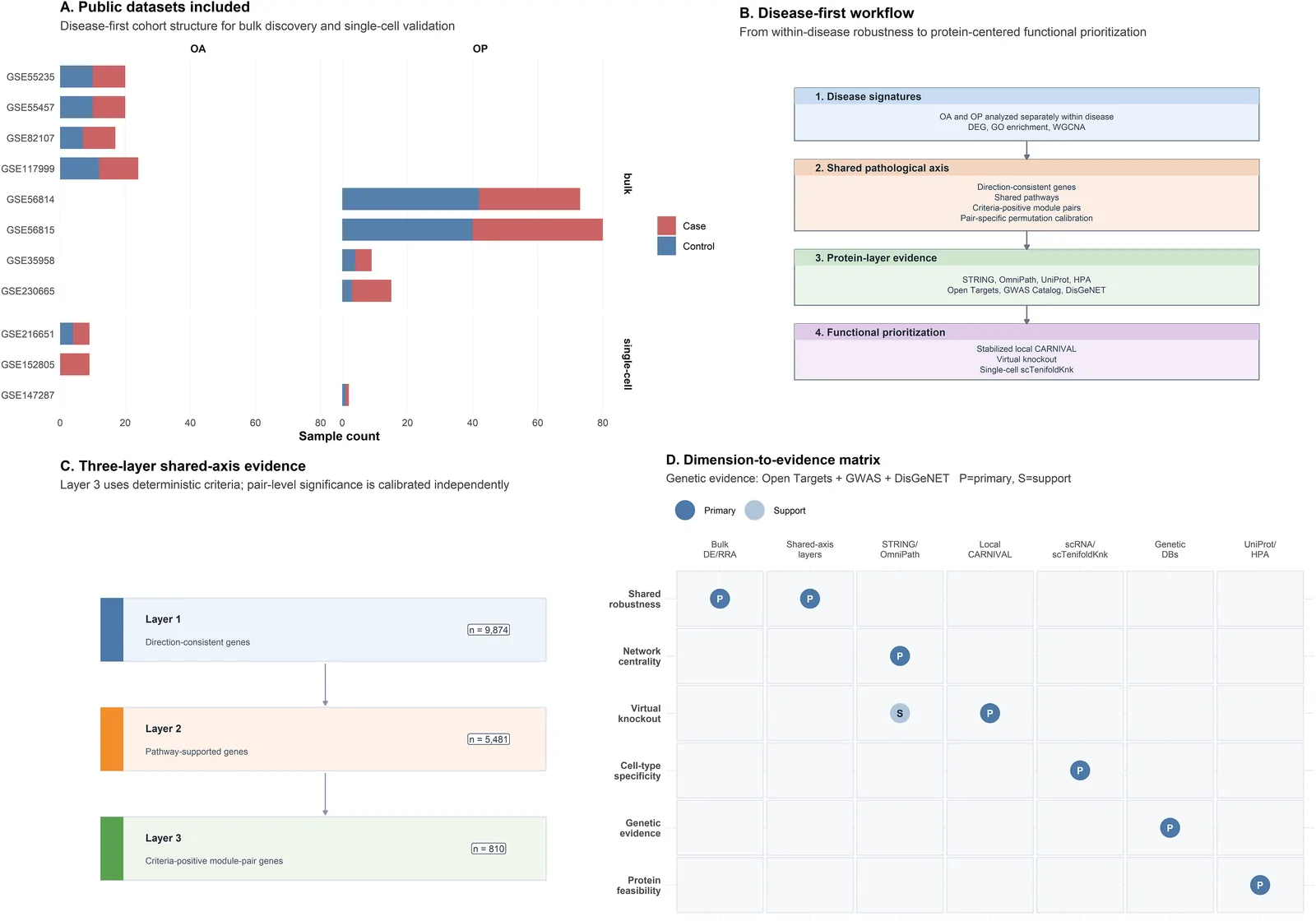
Study design and analytical framework for disease-first multilayer integration of shared osteoarthritis-osteoporosis pathology. (A) Publicly available bulk and single-cell datasets included in the study. Bulk cohorts comprised OA synovial and cartilage datasets and OP monocyte, femoral-bone, and osteogenic-stromal-cell datasets; single-cell datasets were used for cellular-context analysis. (B) Disease-first workflow in which OA and OP were analyzed separately before construction of the shared pathological axis and protein-level prioritization; the single-cell layer additionally included external quality-control sensitivity analysis. (C) Three cumulative evidence layers comprising direction-consistent genes, shared-pathway-supported genes, and genes contained in criteria-positive OA-OP coexpression-module pairs. The three layers contained 9,874, 5,481, and 810 genes, respectively. Pair-specific module correspondence was evaluated using permutation-derived empirical FDR. (D) Evidence sources contributing to shared-disease robustness, network centrality, virtual-knockout evidence, cell-type specificity, genetic evidence, and protein feasibility. OA, osteoarthritis; OP, osteoporosis; DEG, differentially expressed gene; WGCNA, weighted gene coexpression network analysis; HPA, Human Protein Atlas.

All cohorts were processed within disease before any cross-disease integration. OA and OP were modeled separately at the differential-expression and network-construction stages, and cross-disease comparison was introduced only after disease-level signatures or disease-specific network features had been derived (Fig 1B–D).

### OA and OP each exhibited robust disease signatures before shared-axis construction

Independent limma-based differential analyses followed by disease-level robust rank aggregation yielded reproducible signatures for OA and OP (Fig 2A–D). In OA, top-ranked robust genes included SON, TTC3, GRB10, PRRC2C, and LRRFIP1, whereas in OP the top-ranked genes included NCOA1, DOK1, SLC43A3, TMEM14B, INTS6, and PIAS1. Representative narrative-anchor genes in OA were concentrated in antigen presentation, inflammatory signaling, wound response, and matrix- or bone-interface biology, whereas OP anchors were concentrated in immune activity, myeloid signaling, antigen presentation, and bone remodeling.

**Fig 2:**
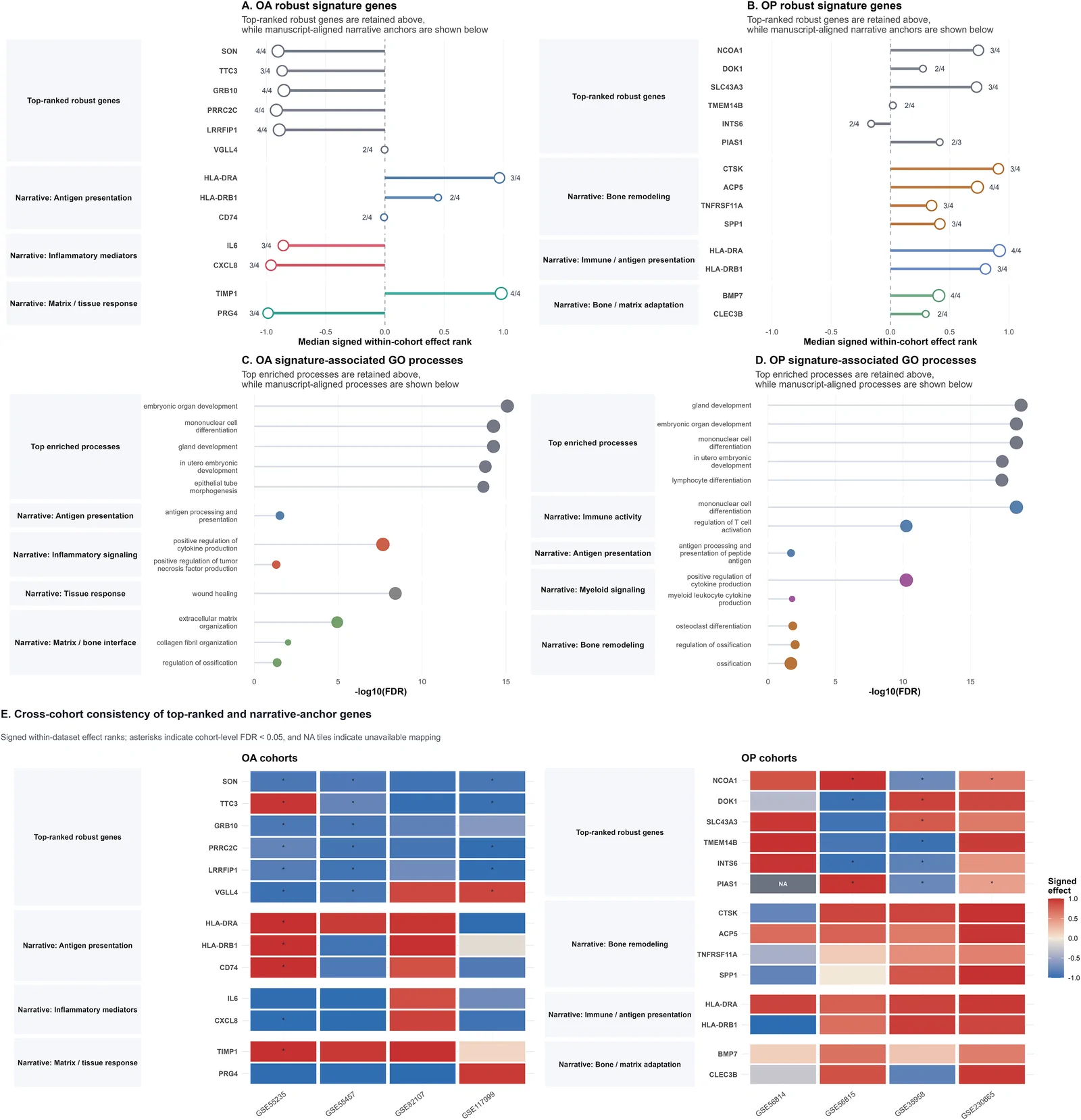
Robust disease signatures and enriched biological programs in osteoarthritis and osteoporosis. (A) OA signature genes prioritized by integration across four OA bulk cohorts. The panel presents the highest-ranked genes and selected genes representing antigen presentation, inflammatory signaling, and matrix or tissue-response processes. (B) OP signature genes prioritized across four OP bulk cohorts, together with selected genes representing bone remodeling, immune and antigen-presentation activity, and bone or matrix adaptation. (C) Representative Gene Ontology biological processes enriched in the OA signature. (D) Representative Gene Ontology biological processes enriched in the OP signature. (E) Cross-cohort effects of the displayed genes. Cell color indicates the signed within-dataset effect, asterisks indicate cohort-level FDR < 0.05, and NA indicates unavailable mapping. Complete cohort-level gene and pathway results are provided in the accompanying data files. OA, osteoarthritis; OP, osteoporosis; GO, Gene Ontology; FDR, false discovery rate.

At the functional level, OA robust signatures were enriched in antigen processing and presentation, positive regulation of cytokine production, positive regulation of tumor necrosis factor production, wound healing, extracellular matrix organization, collagen fibril organization, and regulation of ossification (Fig 2C). OP robust signatures were enriched in mononuclear cell differentiation, regulation of T-cell activation, antigen processing and presentation of peptide antigen, positive regulation of cytokine production, myeloid leukocyte cytokine production, osteoclast differentiation, regulation of ossification, and ossification (Fig 2D).

Across individual bulk cohorts, representative OA and OP genes showed recurrent directional behavior in the same overall direction after within-disease integration, although effect magnitude varied by cohort and tissue context (Fig 2E). These results established reproducible disease-level transcriptional signatures before cross-disease comparison.

Exclusion of GSE35958 yielded rank correlations of 0.916 for the OP RRA and 0.895 for the shared axis relative to the primary analysis, with 5/20 and 10/20 primary top-ranked features retained, respectively (S1 Fig and S1 Table). Layer 1 + 2 and Layer 1 + 2 + 3 candidate counts changed from 5,481–5,391 and from 810 to 804, respectively. In the conditional protein-level comparison, rank correlation was 0.918 and 8 of the primary top 10 proteins were retained; HSP90AA1 and RPL7 no longer met the cross-disease direction-consistency criterion.

### A three-layer framework integrated shared OA-OP evidence

Shared OA-OP pathology was defined using three ordered evidence layers (Fig 3A–B). Layer 1 retained genes with concordant direction of dysregulation between OA and OP after disease-level integration. Layer 2 further restricted candidates to genes supported by biological processes enriched in both diseases. The resulting shared pathway space was concentrated in immune and inflammatory activity, antigen presentation, extracellular-matrix organization, collagen-related structure, and bone-remodeling or ossification-related processes (Fig 3B).

**Fig 3:**
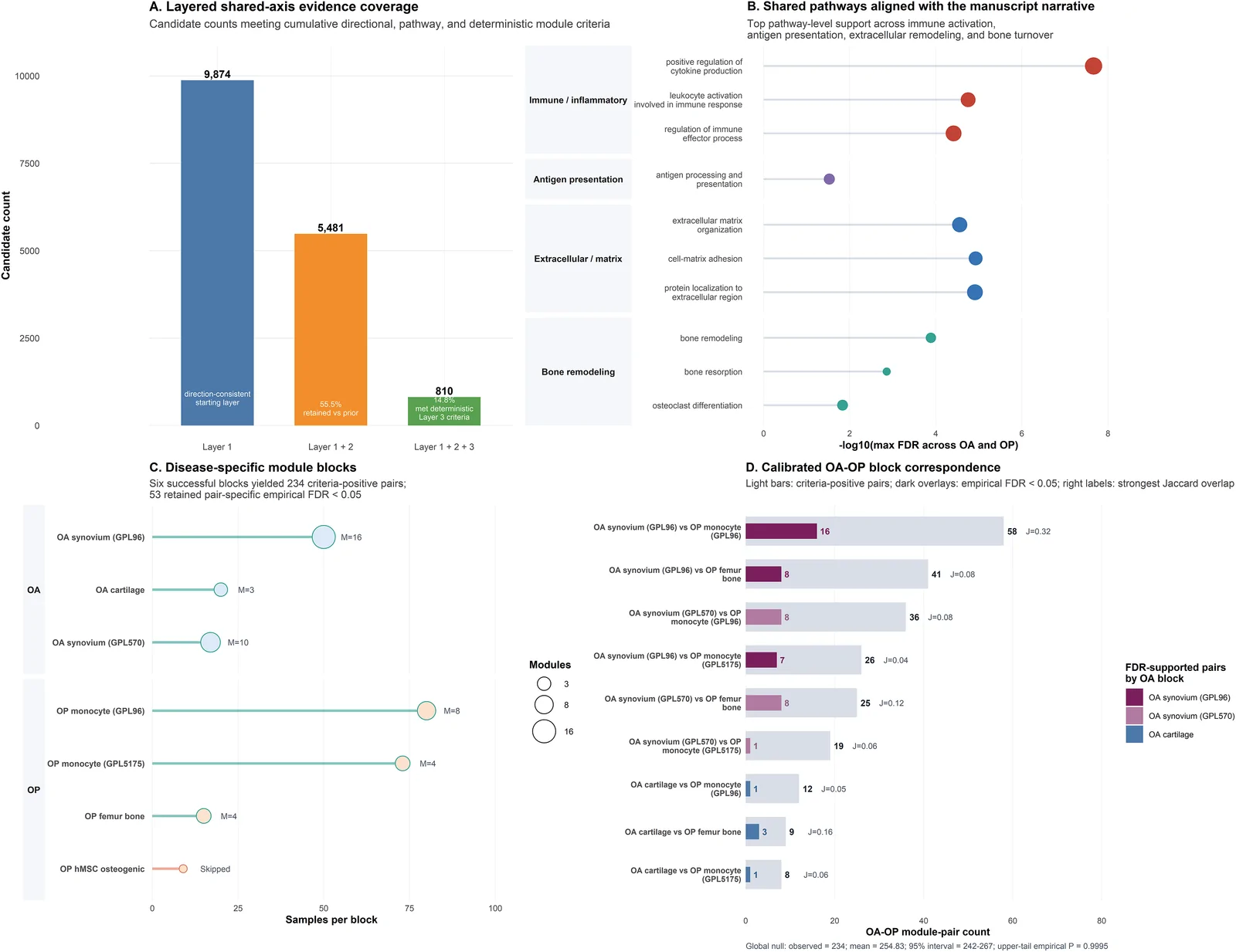
Layered shared-axis evidence and permutation-calibrated OA-OP module correspondence. (A) Numbers of genes meeting the cumulative direction-consistency, shared-pathway, and module-correspondence criteria. Layer 3 contained 810 genes occurring in at least one criteria-positive OA-OP module pair. (B) Representative shared biological processes involving immune activation, antigen presentation, extracellular-matrix remodeling, and bone remodeling. (C) Disease-specific WGCNA blocks and numbers of retained disease-associated modules. Six eligible blocks produced 234 criteria-positive cross-disease module pairs. (D) Numbers of criteria-positive pairs and pairs with empirical FDR < 0.05 in each OA-OP block combination. Fifty-three of 464 tested module combinations had empirical FDR < 0.05. The total criteria-positive count was 234 compared with a null mean of 254.83 and a 95% interval of 242–267. OA, osteoarthritis; OP, osteoporosis; FDR, false discovery rate.

Layer 3 incorporated disease-associated coexpression structure derived independently in OA and OP (Fig 3C). Across 464 tested OA-OP module combinations, 234 pairs met the prespecified deterministic matching criteria (Fig 3D). In 10,000 block-stratified, size-preserving permutations, 53 of these pairs retained pair-specific support at empirical FDR < 0.05, and an independent repeat using a second random seed recovered the same 53 pairs (Fig 3D; S2 Fig; S3 Table). The total number of criteria-positive pairs did not exceed the global null expectation (observed, 234; null mean, 254.83; 95% interval, 242–267; upper-tail empirical P = 0.9995), indicating that module-level evidence was concentrated in selected pair-specific correspondences rather than a globally enriched OA-OP module-matching architecture.

### Protein-level integration prioritized proteins across complementary evidence dimensions

Shared-axis candidates were mapped to proteins and integrated with STRING interaction topology [20], OmniPath signaling priors [21], UniProt annotation [22], Human Protein Atlas tissue support [23], Open Targets evidence [24], DisGeNET evidence [25], and direct GWAS Catalog associations [26] (Fig 4, Table 2). The primary integrated ranking prioritized HLA-DRB1, HSP90AA1, CTSK, RPL7, PRG4, HLA-DRA, CLEC3B, TIMP1, SPP1, and APOE (Fig 4A, Table 2).

**Table 2:** Top 10 prioritized proteins in shared OA-OP pathology.

Rank	Protein	Total score	Primary CARNIVAL status	Shared-disease robustness	Network centrality	Normalized CARNIVAL evidence	Cell-context support	Genetic evidence	Protein feasibility	FDR-supported module pairs
1	HLA-DRB1	0.674	Above model-derived threshold	0.843	0.02	1	0.875	0.516	0.455	2
2	HSP90AA1	0.528	Above model-derived threshold	0.627	0.701	0.556	0.724	0	0.545	3
3	CTSK	0.353	Evaluated, below threshold	0.397	0.009	0	0.639	0.588	1	4
4	RPL7	0.345	Not in local network	1	0.038	0	0.806	0	0.091	2
5	PRG4	0.335	Not in local network	0.606	0	0	1	0.008	0.818	0
6	HLA-DRA	0.327	Evaluated, below threshold	0.598	0.022	0	0.897	0.261	0.455	0
7	CLEC3B	0.322	Not in local network	0.607	0	0	0.543	0.227	0.818	2
8	TIMP1	0.312	Evaluated, below threshold	0.762	0.014	0	0.721	0.014	0.455	0
9	SPP1	0.285	Evaluated, below threshold	0.329	0.05	0	0	0.996	0.455	1
10	APOE	0.262	Evaluated, below threshold	0.264	0.154	0	0	0.85	0.455	0

Primary CARNIVAL status refers to the first evaluable baseline–knockout pair in the prespecified configuration sequence. Normalized CARNIVAL evidence is the 0–1 transformed evidence dimension used in the composite protein score; model-derived CARNIVAL scores are shown in Fig 5. Configuration-level evaluability, model-derived CARNIVAL scores, and stability summaries are provided in S4 Table. Degree-corrected and annotation-removal sensitivity rankings are provided in S7 Table.

**Fig 4:**
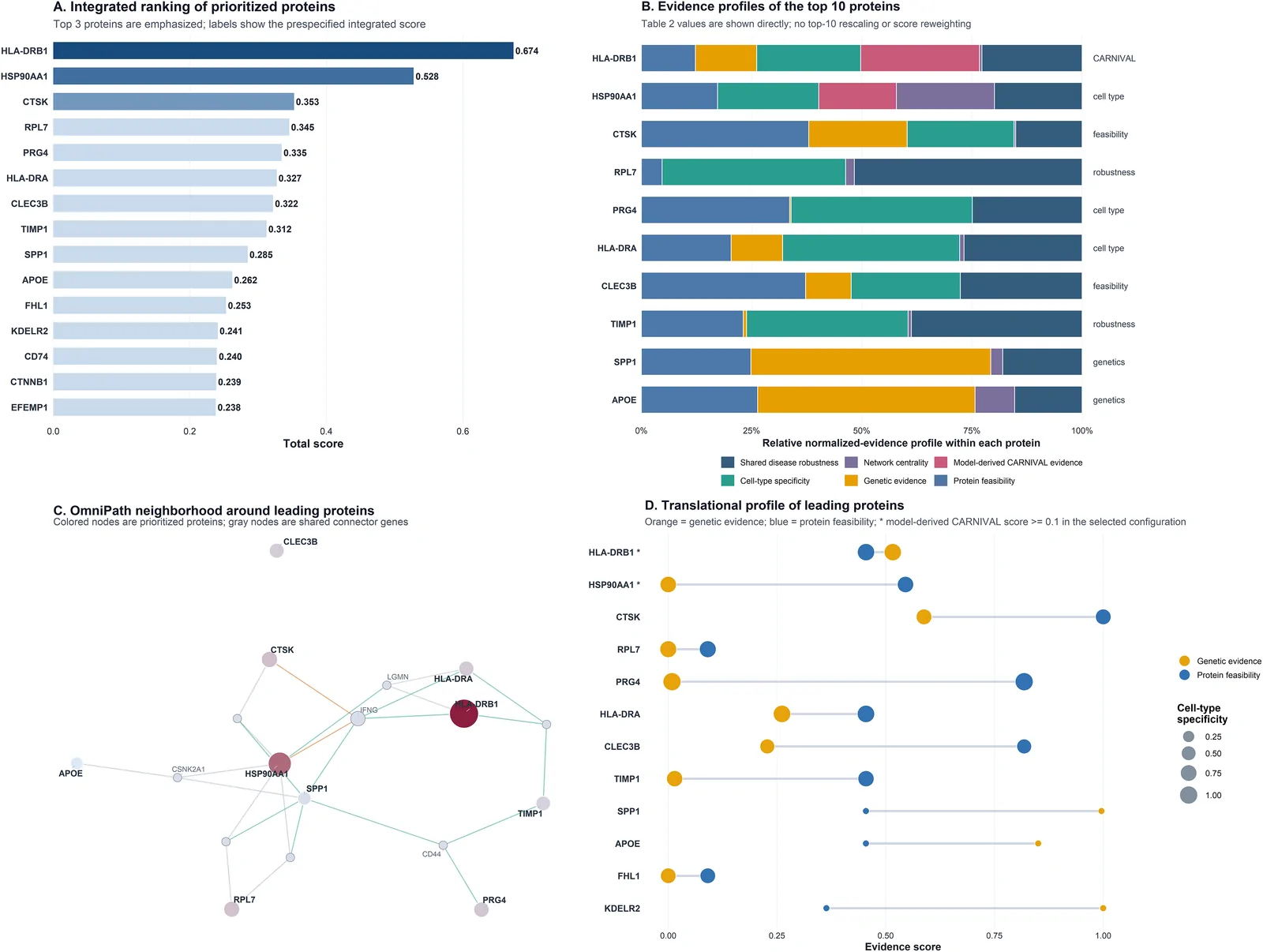
Protein-level evidence integration and prioritization of shared OA-OP candidates. (A) Integrated ranking of the top 15 proteins according to the composite priority score, with the three highest-ranked proteins highlighted. (B) Profiles of the six normalized evidence dimensions for the top 10 proteins. Values correspond to those reported in Table 2, and right-side labels identify the largest evidence dimension for each protein. (C) OmniPath neighborhood of the leading proteins. Colored nodes indicate prioritized proteins, gray nodes indicate shared connector genes, and directed edges indicate curated signaling relationships. (D) Genetic-evidence and protein-feasibility profiles of the leading proteins. Point size represents cell-type specificity, and asterisks identify proteins with model-derived CARNIVAL score of at least 0.1 in the selected CARNIVAL configuration. OA, osteoarthritis; OP, osteoporosis. Degree- and annotation-aware sensitivity results are shown in S6 Fig and S7 Table.

The evidence composition of the leading proteins differed across the six scoring dimensions (Fig 4B). HLA-DRB1 ranked first overall (0.674), with the model-derived CARNIVAL component contributing its largest normalized evidence value [27]. HSP90AA1 ranked second (0.528) and was dominated by cell-context support. CTSK ranked third (0.353) and was dominated by protein feasibility, reaching the maximum feasibility score in the summary table. RPL7 and TIMP1 were dominated by shared-disease robustness, PRG4 and HLA-DRA by cell-context support, CLEC3B by protein feasibility, and SPP1 and APOE by genetic evidence (Table 2). Fig 4C places the leading proteins within their OmniPath neighborhood, whereas Fig 4D compares their genetic-evidence and protein-feasibility profiles.

Among the top 10 proteins, HLA-DRB1, HSP90AA1, CTSK, RPL7, CLEC3B, and SPP1 occurred in at least one FDR-supported module pair, with supported-pair counts of 2, 3, 4, 2, 2, and 1, respectively. No FDR-supported module-pair correspondence was identified for PRG4, HLA-DRA, TIMP1, or APOE.

Direct external evidence further supported the ranking. Among the top 200 proteins evaluated, 49 showed nonzero OA- or OP-relevant evidence in GWAS Catalog and 8 retained nonzero disease-association support in DisGeNET. Within the leading proteins, SPP1 and APOE showed the strongest genetic-evidence scores, whereas CTSK, PRG4, and CLEC3B remained prominent because protein-feasibility and context-related evidence remained high after integration.

The primary integrated score correlated with STRING degree (Spearman ρ = 0.476, P < 2.2 × 10^−16^). Degree correction retained 9 of the primary top 10 and 165 of the top 200 proteins, with a rank correlation of ρ = 0.882 among the primary top 200. Omission of network centrality retained 9 of the top 10 and 190 of the top 200 proteins. HLA-DRB1 and HSP90AA1 remained ranked first and second in both analyses. Among the externally evaluated top 200 proteins, the primary score correlated with generic UniProt/HPA annotation count (ρ = 0.388, P = 1.36 × 10^−8^); after omission of protein feasibility, this correlation decreased to ρ = 0.106 (P = 0.133. Omission of both genetic evidence and protein feasibility retained 7 of the top 10 and 185 of the top 200 proteins, with SPP1 and APOE moving to ranks 34 and 38, respectively (S6 Fig and S7 Table).

### Composition-aware analyses qualified recurrent MHC-II and ribosomal signals

Cell-composition sensitivity analysis was performed in four eligible mixed-tissue cohorts: GSE55457, GSE82107, GSE117999, and GSE230665. None of the 40 population-by-cohort comparisons remained significant after global false-discovery-rate correction (S5A Fig; S6 Table). After adjustment for the first two principal components of the inferred abundance scores, 36 of 40 candidate–cohort effects had the same sign before and after adjustment (S5B Fig). Direction was retained in all four cohorts for APOE, HLA-DRA, HSP90AA1, PRG4, RPL7, SPP1, and TIMP1; retention was observed in three of four cohorts for HLA-DRB1 and CLEC3B and in two of four cohorts for CTSK. Fifty of 400 residual candidate–population associations remained significant at global FDR < 0.05 (S5C Fig), indicating that some candidate signals continued to track inferred cellular abundance within disease groups.

Adjustment for matched transcriptional programs retained the direction of 24 of 27 evaluable candidate–cohort effects (S5D Fig; S6 Table). Direction was retained for HLA-DRA in seven of seven cohorts, HLA-DRB1 in five of seven, HSP90AA1 in six of seven, and RPL7 in six of six. The median absolute adjusted-to-unadjusted effect ratios were 0.483 for HLA-DRA, 0.584 for HLA-DRB1, 1.117 for HSP90AA1, and 0.850 for RPL7. Thus, the MHC-II candidates showed greater attenuation after adjustment for the corresponding transcriptional program, whereas the HSP90AA1 and RPL7 effects were generally more stable.

### Local CARNIVAL scores showed configuration-stable and configuration-sensitive model-derived profiles

Stabilized local CARNIVAL virtual knockout was evaluated across the prespecified local-network configurations (Fig 5). In the primary configuration, HLA-DRB1 had the highest model-derived CARNIVAL score (2.25), followed by HSP90AA1 (1.25); both exceeded the prespecified threshold of 0.1.

**Fig 5:**
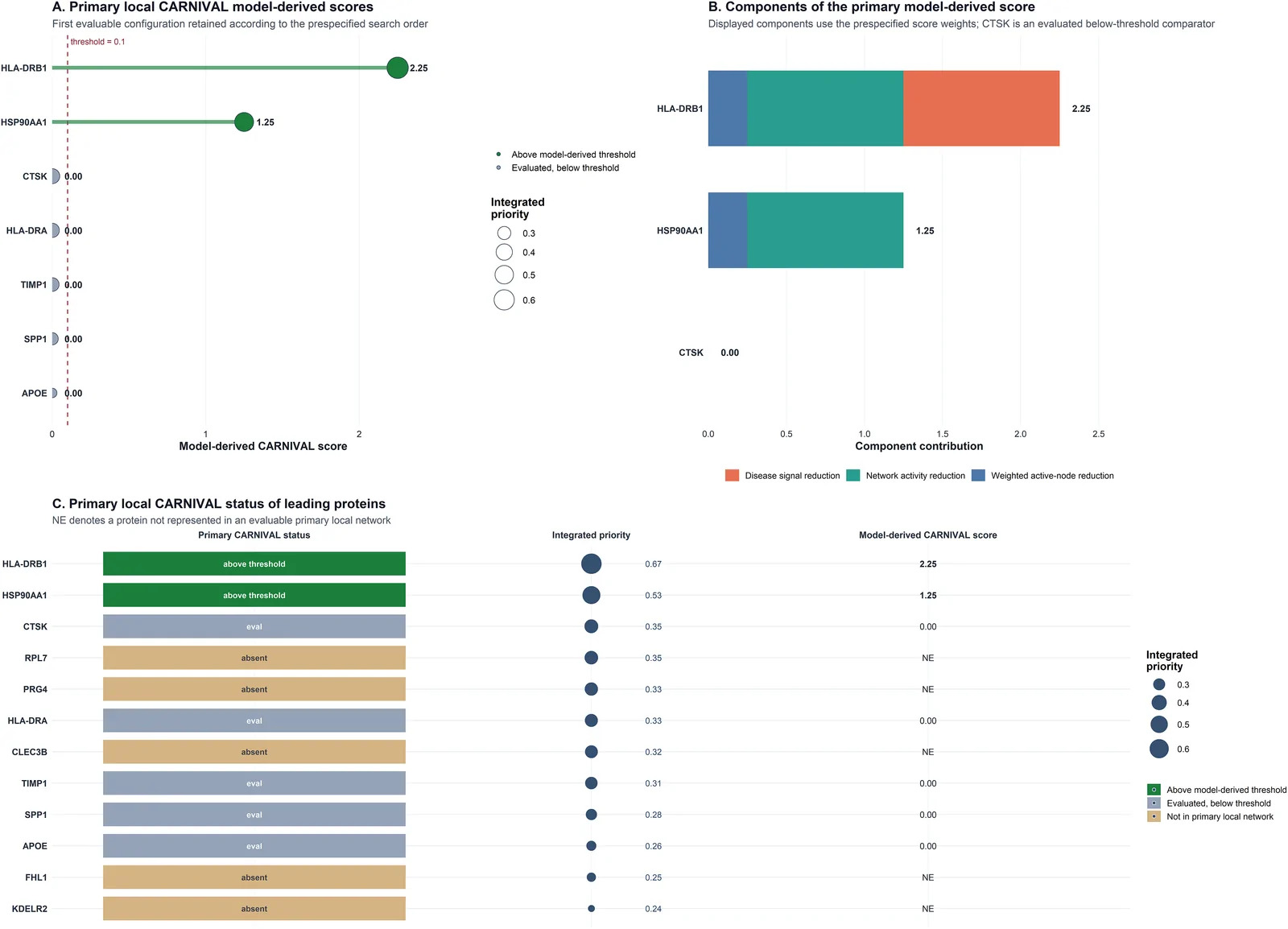
Virtual-knockout results in the selected local CARNIVAL configuration. (A) Raw model-derived CARNIVAL score for proteins with evaluable baseline and knockout solutions. The dashed line indicates the exceeding the prespecified model-derived threshold of 0.1. (B) Contributions from disease-signal reduction, network-activity reduction, and weighted active-node reduction to the raw model-derived CARNIVAL scores of HLA-DRB1 and HSP90AA1; CTSK is shown as an evaluated below-threshold comparator. (C) CARNIVAL status, integrated priority, and raw model-derived CARNIVAL score of the leading proteins. NE indicates that no evaluable local-network result was obtained. Configuration-level results across six local-network settings are provided in S3 Fig and S4 Table.

Model-derived score decomposition attributed the HLA-DRB1 and HSP90AA1 scores to reductions in disease signal, network activity, and active-node burden, whereas CTSK had a score of 0 (Fig 5B). Among the remaining leading proteins, some were evaluable with scores below the prespecified threshold, whereas others were absent from an evaluable local network (Fig 5C).

Configuration-sensitivity analysis evaluated 20 candidate proteins across six combinations of measurement-set size and connector limit, yielding 120 protein-configuration combinations (S3 Fig and S4 Table). Sixty-two combinations produced evaluable baseline and knockout solutions, 54 were non-evaluable because the candidate was absent from the reconstructed local network, and four failed the minimum-input precheck. HLA-DRB1 exceeded the model-derived threshold in all four evaluable configurations, with an invariant model-derived CARNIVAL score of 2.25; the two configurations using four measurements were not evaluable because the knockout network did not retain the minimum required input. HSP90AA1 was evaluable in all six configurations and exceeded the model-derived threshold in four, with a score of 1.25 under the measurement-set sizes of eight and six but a score of 0 under both four-measurement configurations. CTSK was evaluable in all six configurations but had a model-derived CARNIVAL score of 0 throughout. The four configurations using measurement-set sizes of eight or six produced identical model-derived score profiles and rankings (pairwise Spearman ρ = 1.0). Rank correlation was not defined for the two four-measurement configurations because all evaluable model-derived CARNIVAL score were zero.

### Matched-null calibration identified context-specific single-cell perturbation outliers

Single-cell analysis characterized candidate expression and calibrated perturbation across four disease-relevant cellular compartments (Fig 6, Table 3). Candidate expression was summarized across OA fibroblasts, OA macrophages, OP bone marrow mesenchymal stromal cells, and OP osteoclast precursors. The raw scTenifoldKnk perturbation scores were then calibrated against 200 expression- and wild-type network-degree-matched control genes for each of the 39 evaluable candidate-compartment pairs. Fig 6C shows the resulting matched-null Z scores, whereas Fig 6D summarizes the leading calibrated pairs separately for OA and OP. Full matched-null distributions and empirical significance results are provided in S4 Fig and S5 Table.

**Table 3:** Primary and external quality-control sensitivity results for single-cell candidate-compartment perturbation pairs.

Disease	Dataset	Compartment	Primary analysis	Cells after QC	QC sensitivity analysis
OA	GSE216651	Macrophage	10 evaluable; CTSK, CLEC3B, TIMP1, PRG4, and HSP90AA1 supported	6,983	11 evaluable; RPL7, TIMP1, HLA-DRB1, HSP90AA1, and HLA-DRA supported
OA	GSE216651	Fibroblast	10 evaluable; RPL7 supported	24,830	10 evaluable; CLEC3B, CTSK, and FHL1 supported
OP	GSE147287	BM-MSC	10 evaluable; CLEC3B supported	2,444	9 evaluable; no globally FDR-supported pair
OP	GSE147287	Osteoclast precursor	9 evaluable; TIMP1 and RPL7 supported	128	9 evaluable; MMP9 and CTSK supported

Primary and quality-controlled perturbation scores were calibrated separately against 200 control genes matched on mean log-normalized expression and wild-type weighted out-degree. One-sided empirical P values used the plus-one correction and were adjusted across the 39 evaluable pairs within each analysis using the Benjamini-Hochberg method. All quality-controlled pairs listed in the table had global FDR = 0.019. Complete pair-level results and sample-level quality-control counts are provided in S8 Table.

**Fig 6:**
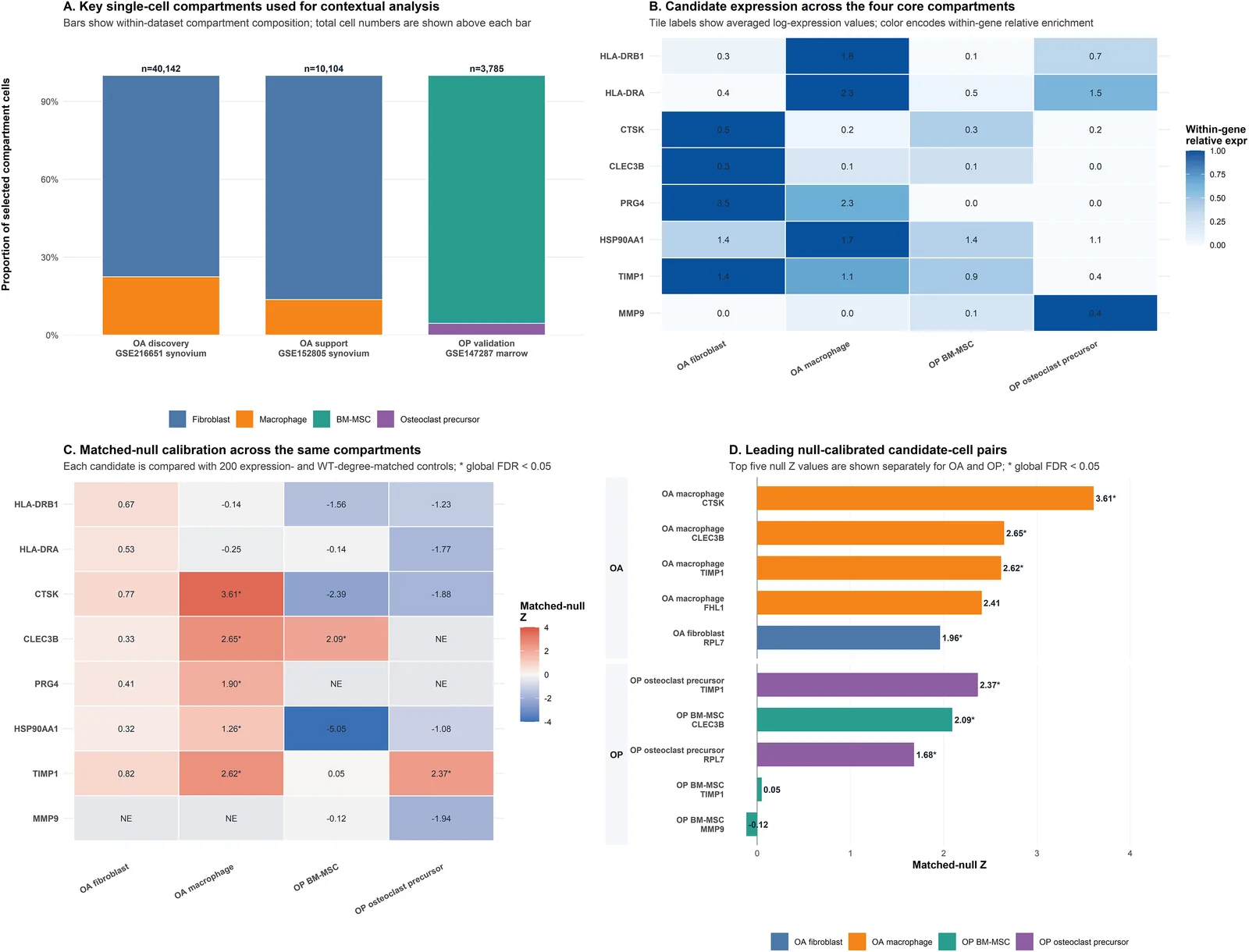
Primary single-cell localization and matched-null calibration of perturbation scores. (A) Cellular composition of the OA and OP single-cell datasets used for contextual analysis. (B) Candidate expression in OA fibroblasts, OA macrophages, OP bone marrow mesenchymal stromal cells, and OP osteoclast precursors. Tile labels show mean log-normalized expression, and color indicates within-gene relative expression. (C) Matched-null Z scores for 39 evaluable candidate-compartment pairs. Each observed score was compared with scores from 200 control genes matched on mean log-normalized expression and wild-type weighted out-degree within the corresponding 600- or 601-gene network stratum. Asterisks indicate global FDR < 0.05, and NE indicates that no evaluable result was obtained. (D) Five leading calibrated pairs shown separately for OA and OP. One-sided empirical P values used the plus-one correction and were adjusted across all 39 evaluable pairs. OA, osteoarthritis; OP, osteoporosis; BM-MSC, bone marrow mesenchymal stromal cell; FDR, false discovery rate. External quality-control sensitivity results, including cell-retention statistics and comparison with the primary candidate-compartment findings, are provided in S7 Fig and S8 Table.

Across the 39 evaluable candidate-compartment pairs, 11 had one-sided empirical P values below 0.05 and nine remained supported after Benjamini-Hochberg correction across all 39 tests. In OA macrophages, the supported pairs were CTSK (null Z = 3.61, q = 0.024), CLEC3B (Z = 2.65, q = 0.024), TIMP1 (Z = 2.62, q = 0.024), PRG4 (Z = 1.90, q = 0.024), and HSP90AA1 (Z = 1.26, q = 0.024). In OA fibroblasts, RPL7 was retained (Z = 1.96, q = 0.024). Although FHL1 showed a positive OA macrophage signal (Z = 2.41, empirical P = 0.0249), it did not remain significant after global correction (q = 0.097).

In the OP compartments, CLEC3B was supported in bone marrow mesenchymal stromal cells (null Z = 2.09, q = 0.024). TIMP1 (Z = 2.37, q = 0.043) and RPL7 (Z = 1.68, q = 0.024) were supported in osteoclast precursors. Several candidates with comparatively high raw perturbation scores, including MMP9, HLA-DRA, HLA-DRB1, and CTSK in OP compartments, did not exceed their matched-null backgrounds after global correction. These comparisons showed that several high raw perturbation scores were not outliers relative to expression- and network-degree-matched control genes.

Restricting the null distribution to the 100 closest matched control genes retained the same nine globally FDR-supported pairs and produced concordant significance classifications for all 39 evaluated pairs. Matching distances varied among candidate-compartment pairs. The median matching distances for OA macrophage PRG4 and HSP90AA1 were 2.98 and 2.21, respectively.

### External quality-control sensitivity analysis qualified cell-compartment assignments

External quality control retained 125,090 of 161,470 cells (77.47%). The retained cell counts were 76,655 of 105,786 for GSE216651, 34,267 of 36,918 for GSE152805, and 14,168 of 18,766 for GSE147287. After quality control, the four perturbation compartments contained 24,830 OA fibroblasts, 6,983 OA macrophages, 2,444 OP bone marrow mesenchymal stromal cells, and 128 OP osteoclast precursors. Dataset- and sample-level retention statistics are provided in S7 Fig and S8 Table.

The quality-controlled analysis produced 39 evaluable candidate-compartment pairs, of which 10 remained supported at global FDR < 0.05. Supported pairs comprised CLEC3B, CTSK, and FHL1 in OA fibroblasts; RPL7, TIMP1, HLA-DRB1, HSP90AA1, and HLA-DRA in OA macrophages; and MMP9 and CTSK in OP osteoclast precursors. No pair remained globally supported in OP bone marrow mesenchymal stromal cells. Among the 38 pairs evaluable in both analyses, correlations between the primary and quality-controlled results were 0.105 for raw perturbation scores and 0.015 for matched-null Z scores. HSP90AA1 and TIMP1 in OA macrophages were the two primary pairs retained in the same compartment, whereas CTSK, CLEC3B, and RPL7 remained supported but shifted between compartments.

When the quality-controlled cell-context evidence was substituted in a ranking sensitivity analysis, all 10 primary top-ranked proteins and 19 of the primary top 20 proteins were retained. The overall rank correlation was greater than 0.999, and HLA-DRB1 and HSP90AA1 remained ranked first and second. Thus, external quality control materially affected pair-level cellular localization but had negligible influence on the overall protein-prioritization hierarchy.

## Discussion

This study presents a disease-first, multilayer framework for investigating shared OA-OP pathology from public datasets. Overall, the findings suggest that OA and OP may converge through interconnected inflammatory, extracellular matrix-remodeling, immune, and bone-homeostasis programs rather than representing entirely unrelated disease processes. This interpretation is supported by the convergence of disease-specific signatures, layered shared-axis definition, protein-level evidence integration, virtual knockout analysis, and single-cell contextualization accompanied by matched-null calibration and external quality-control sensitivity testing.

Our findings are broadly consistent with the evolving view that OA and OP are not merely opposing skeletal phenotypes, but may share inflammation-, immune-, stress-, and remodeling-related mechanisms in ageing musculoskeletal tissues. OA is increasingly recognized as a whole-joint disorder involving synovial inflammation, cartilage degeneration, subchondral bone remodeling, matrix turnover, cellular senescence, and impaired autophagy, whereas OP is driven by imbalanced bone remodeling under the influence of osteoimmune activation, oxidative stress, and altered osteoblast–osteoclast coupling. In this context, the convergence of OA and OP signatures on antigen presentation, cytokine-related regulation, extracellular matrix organization, collagen-associated processes, osteoclast differentiation, and ossification is biologically plausible. The prioritized proteins identified in the present study also fit this background. HLA-DRB1 and HLA-DRA reflect antigen-presentation and immune-activation programs [28]; CTSK and TIMP1 are closely related to bone resorption and matrix remodeling [29,30]; PRG4, CLEC3B, and SPP1 point to extracellular matrix and tissue-repair biology [31–33]; and APOE may connect lipid handling, inflammation, and joint–bone homeostasis [16]. Therefore, rather than indicating a simple overlap between two disease gene lists, the present results support a shared osteoimmune–matrix-remodeling axis linking OA and OP across joint and bone compartments.

Local virtual knockout assigned the two highest model-derived model-derived CARNIVAL score to HLA-DRB1 and HSP90AA1. Across evaluable configurations, the HLA-DRB1 score was more stable than the HSP90AA1 score. HLA-DRB1 encodes a major histocompatibility complex class II molecule involved in antigen presentation and CD4-positive T-cell activation [28]. Its prioritization suggests that immune recognition and antigen-presentation programs may contribute to the shared OA-OP state, particularly in synovial inflammatory and marrow-remodeling contexts. This interpretation is consistent with the increasing appreciation that osteoimmune interactions can influence both pathological bone resorption and inflammatory joint remodeling [34]. However, HLA-DRB1 should not be interpreted as a direct therapeutic target at this stage. Instead, it may represent an immune-context marker or model-prioritized network node that captures antigen-presentation activity within the shared disease network. HLA-DRB1 retained the same rescue score of 2.25 in all four configurations in which its knockout network remained evaluable. The two four-measurement configurations were non-evaluable because the knockout network did not meet the minimum-input requirement. HLA-DRB1 therefore showed the greatest configuration stability among candidates exceeding the model-derived threshold.

HSP90AA1 represents a different type of candidate. As a molecular chaperone, HSP90AA1 participates in proteostasis, stress adaptation, autophagy-related regulation, and stabilization of multiple signaling proteins [35,36]. It retained a rescue score of 1.25 under the four configurations using measurement-set sizes of eight or six but had a score of 0 under the two four-measurement configurations. This configuration dependence limits the evidence for a stable model-based network signal. Impaired autophagy, oxidative stress, inflammation, and senescence are implicated in OA progression, whereas heat-shock proteins and chaperone-mediated mechanisms also influence osteoblast and osteoclast biology [37,38]. HSP90AA1 may therefore represent a proteostasis- and stress-response component of the shared OA-OP program. Its loss of cross-disease direction consistency after exclusion of GSE35958 further identified it as both cohort-sensitive and configuration-sensitive. HSP90AA1 consequently remains a secondary candidate for experimental validation.

A major strength of this study is its disease-first order of inference. Instead of directly intersecting DEGs or pooling heterogeneous OA and OP datasets, we first derived disease-specific signatures and network features within each condition, and introduced cross-disease comparison only after within-disease evidence had been established. This strategy reduces the risk that apparent OA-OP convergence is driven by tissue composition or platform heterogeneity. The shared axis integrated concordant dysregulation, shared pathway enrichment, and module-correspondence evidence. Permutation calibration indicated that statistical support within the module layer was concentrated in selected pair-specific correspondences rather than a global excess across all OA-OP module combinations. These findings support localized cross-disease module correspondence but not global enrichment across all OA-OP module combinations.

By integrating protein-interaction, signaling, annotation, tissue-support, disease-association, and genetic evidence, the workflow further moved from transcript-level overlap to protein-level prioritization. The resulting candidates represented complementary biological programs, including antigen presentation and immune activation, signaling-network stabilization, bone resorption, extracellular matrix remodeling, and immune-bone homeostasis. Local CARNIVAL analysis added a model-based functional prioritization layer. HLA-DRB1 showed the highest and most configuration-stable model-derived CARNIVAL score, whereas the high primary score of HSP90AA1 was not retained under the two smallest measurement configurations. Candidates such as CTSK remained highly ranked through complementary protein-feasibility, disease-relevance, and cell-context evidence despite having a CARNIVAL score of 0.

The primary single-cell analysis provided cellular context for the prioritized proteins, but external quality-control sensitivity analysis showed that precise candidate-compartment assignments were not uniformly stable. HSP90AA1 and TIMP1 retained support in OA macrophages, whereas CTSK, CLEC3B, and RPL7 remained supported but shifted between compartments. Five of the six genes supported in the primary analysis retained evidence in at least one quality-controlled compartment, but only two of the nine primary candidate-compartment pairs were retained without a change in cellular context. In contrast, recalculation of the cell-context dimension retained the complete primary top 10 protein set and preserved HLA-DRB1 and HSP90AA1 as the two leading candidates. The single-cell findings therefore support candidate-level cellular relevance but do not establish fixed disease-associated cell-type localization.

The moderate association between the integrated score and STRING degree indicated that network topology contributed to the overall ranking. However, HLA-DRB1 and HSP90AA1 remained ranked first and second when centrality was degree-corrected or omitted, consistent with substantial support from the other evidence dimensions. In contrast, removal of genetic evidence and protein feasibility moved SPP1 and APOE to ranks 34 and 38, respectively, showing that their prioritization depended more strongly on these external evidence sources. The leading proteins therefore differed in both the composition and robustness of their supporting evidence.

The recurrent prioritization of MHC-II and ribosomal proteins requires cautious interpretation. Most candidate–cohort effects retained their direction after adjustment for inferred cellular abundance, arguing against composition as the sole explanation for the bulk signals. Nevertheless, residual associations with inferred cell populations remained for several candidates, and HLA-DRA and HLA-DRB1 were attenuated after adjustment for the broader MHC-II transcriptional program. These proteins may therefore reflect both disease-associated immune activity and variation in antigen-presenting-cell abundance or activation state. RPL7 retained its direction in all evaluable cohorts after ribosomal-program adjustment, but its biological interpretation remains linked to general translational activity rather than a disease-specific regulatory function. Accordingly, the MHC-II and ribosomal candidates are best regarded as context-sensitive components of the shared axis rather than confirmed OA–OP-specific regulators.

Overall, this study supports a shared osteoimmune–matrix-remodeling protein axis linking OA and OP, providing candidates for future validation.

## Limitation

Several limitations should also be considered. First, although the configuration-sensitivity analysis evaluated score stability across all six prespecified local-network settings, the virtual-knockout results remain dependent on the OmniPath prior, the selected biological output panels, and the local-network representation. HLA-DRB1 was non-evaluable under the two smallest measurement configurations because its knockout network failed the minimum-input requirement, whereas the HSP90AA1 score decreased to 0 under these settings. These dependencies limit the CARNIVAL results to computational functional prioritization and require experimental evaluation of the predicted network-state changes.

Second, despite the disease-first design, the public datasets remain heterogeneous in tissue source, platform, and cohort composition, and one OP WGCNA block was excluded because stable module construction could not be justified. In addition, permutation calibration did not show a global excess of criteria-positive module pairs over the size-preserving null, although 53 individual pairs retained empirical FDR < 0.05. The module analysis therefore supported 53 pair-specific correspondences but did not demonstrate global enrichment of OA-OP module similarity. Exclusion of GSE35958 preserved overall rank concordance and Layer 1 + 2/3 candidate counts but altered several upper-ranked features, indicating that HSP90AA1 and RPL7 were sensitive to cohort composition.

Third, the single-cell analyses remain computational and should be interpreted as contextual support rather than experimental validation. External quality control removed low-complexity cells, cells with excessive mitochondrial transcript fractions, and predicted doublets, but the candidate-compartment results remained sensitive to the retained cellular composition. Only two primary pairs remained supported in the same compartment, although five of the six primary supported genes retained evidence in at least one compartment. Ambient-RNA correction could not be applied consistently because unfiltered droplet matrices containing empty droplets were not available for all datasets. The limited number of OP osteoclast precursors and variation in sample-level cell retention further constrain compartment-specific interpretation. Independent single-cell datasets and experimental perturbation studies are therefore required to confirm the cellular localization of these candidates. The bulk composition analysis was restricted to four eligible mixed-tissue cohorts and relied on transcriptome-derived abundance estimates rather than directly measured cell fractions. These adjustments cannot fully separate changes in cell abundance from changes in cellular activation state, and residual composition-related confounding may therefore remain.

Fourth, the protein ranking was influenced by network topology and the coverage of public annotation resources. Although HLA-DRB1 and HSP90AA1 remained the leading candidates in degree-aware analyses, SPP1 and APOE were more sensitive to removal of genetic and protein-feasibility evidence. These candidate-specific differences require validation using evidence independent of the resources included in the prioritization framework.

## Conclusion

This study supports a disease-first, multilayer framework for investigating shared biology linking osteoarthritis and osteoporosis. The results suggest that OA and OP may converge through overlapping inflammatory, extracellular matrix-remodeling, immune, and bone-homeostasis programs rather than reflecting simple coexistence of two entirely independent disorders. By preserving disease-specific structure before cross-disease comparison and then integrating protein-level evidence with local virtual knockout and single-cell contextual analysis with matched-null calibration and external quality-control sensitivity testing, we prioritized a focused set of shared candidate proteins for follow-up.

Within this framework, HLA-DRB1 and HSP90AA1 had the highest model-derived CARNIVAL scores in the selected directed prior-knowledge network, whereas CTSK, PRG4, CLEC3B, SPP1, TIMP1, and APOE remained notable because complementary evidence from biological plausibility, protein feasibility, genetic support, or cellular context converged on them. These findings do not by themselves establish definitive causal regulators of shared OA-OP pathology, but they provide a biologically informed shortlist and a practical analytical framework for future mechanistic and translational validation.

## Methods

### Ethics statement

This study analyzed only publicly available, de-identified transcriptomic datasets obtained from GEO (GSE55235, GSE55457, GSE82107, GSE117999, GSE56814, GSE56815, GSE35958, GSE230665, GSE216651, GSE152805, and GSE147287) together with CELLxGENE reference resources. No new participant recruitment, intervention, or biospecimen collection was performed by the authors, and no directly identifiable participant information was accessed. Therefore, no new informed consent was obtained for this secondary analysis.

### Public datasets and overall analytical design

This study was a secondary reanalysis of publicly available, de-identified transcriptomic datasets; no new transcriptomic or participant-level data were generated. Public transcriptomic datasets were collected from GEO. OA bulk cohorts included GSE55235, GSE55457, GSE82107, and GSE117999, whereas OP bulk cohorts included GSE56814, GSE56815, GSE35958, and GSE230665. Single-cell datasets used for contextual validation were GSE216651, GSE152805, and GSE147287, with CELLxGENE used as a reference resource for cell-type interpretation. The analytical design followed a disease-first strategy: OA and OP were modeled separately before any cross-disease integration. Cohorts from different platforms or tissue sources were therefore retained as independent analytical units and were integrated only after disease-level ranking or network construction, rather than by direct early-stage pooling of heterogeneous matrices.

### Differential analysis and disease-signature construction

Each bulk cohort was modeled independently using limma. Cohort-specific gene rankings were integrated separately within OA and OP by robust rank aggregation [39], and Gene Ontology biological-process enrichment was used to characterize the resulting disease-level signatures [40]. Sensitivity to the small OP stromal cohort was assessed by repeating the OP integration after exclusion of GSE35958. Probe mapping, rank-construction rules, enrichment thresholds, and leave-one-dataset-out comparison procedures are detailed in S1 Text.

### Bulk cell-composition and transcriptional-program sensitivity analysis

Cell-composition sensitivity analysis was conducted in four eligible mixed-tissue cohorts with available case–control contrasts and informative abundance estimates: GSE55457, GSE82107, GSE117999, and GSE230665. MCP-counter was used to estimate ten immune and stromal population scores from gene-level expression matrices. The ten leading candidate genes were removed from the marker input before abundance estimation. Samples without finite expression measurements were excluded before analysis; four such samples were excluded from GSE117999, leaving 10 cases and 10 controls. Population scores were standardized within each cohort, and case–control differences were estimated using empirical-Bayes linear models. Benjamini–Hochberg correction was applied across the 40 population-by-cohort comparisons.

Candidate-expression effects were re-estimated after adjustment for the first two principal components of the standardized population-score matrix. Directional stability was defined as retention of the sign of the case–control coefficient before and after adjustment. Residual candidate–population associations were evaluated using Spearman correlation after removing the group effect from both variables, with global false-discovery-rate correction across 400 candidate–population comparisons.

To distinguish candidate-specific associations from broader transcriptional activity, matched gene-program scores were calculated as the mean gene-wise standardized expression of Gene Ontology-defined MHC-II antigen-presentation, ribosomal, and protein-folding gene sets. The evaluated candidate was excluded from its corresponding program. HLA-DRA and HLA-DRB1 were adjusted for the MHC-II program, RPL7 for the ribosomal program, and HSP90AA1 for the protein-folding program. Effect direction and magnitude were compared before and after program-score adjustment across all evaluable cohort–candidate combinations.

### Shared pathological axis and disease-associated module matching

The shared OA-OP axis comprised three ordered evidence layers. For each mapped gene, cohort-specific logFC values were averaged separately within OA and OP. Layer 1 required identical signs for the resulting OA and OP mean effects: positive means in both diseases denoted concordant upregulation, whereas negative means denoted concordant downregulation; genes with opposing signs were excluded. Direction was therefore determined from within-cohort case-control effects rather than by directly comparing expression intensities across platforms. Layer 2 required support from biological processes enriched in both diseases. Layer 3 incorporated correspondence between disease-associated OA and OP coexpression modules constructed using WGCNA within tissue- and platform-consistent blocks [41]. Criteria-positive module pairs were identified by shared-pathway-gene overlap, functional-term overlap, or a joint gene-overlap and Jaccard criterion. Pair-specific statistical support was evaluated using block-stratified permutation calibration with false-discovery-rate correction. Complete WGCNA settings, module-retention criteria, matching thresholds, and permutation procedures are provided in S1 Text.

### Protein-level evidence integration and final prioritization

Shared-axis candidates were mapped to proteins and evaluated across six evidence dimensions: shared-disease robustness; interaction-network centrality based on STRING [20]; model-derived virtual-knockout support based on OmniPath and CARNIVAL [21,27]; cell-context support; genetic evidence from Open Targets, GWAS Catalog, and DisGeNET [24–26]; and protein feasibility based on UniProt and Human Protein Atlas annotations [22,23]. The dimensions were independently normalized and combined using prespecified weights because no labeled outcome was available for empirical weight estimation. Direct GWAS Catalog and DisGeNET evaluation was restricted to the first 200 proteins after protein-layer integration. Definitions, normalization rules, and weights for all six dimensions are reported in S1 Text and S2 Table.

Sensitivity to network hubness and annotation density was evaluated using degree-corrected centrality, omission of network centrality, and omission of selected external-evidence dimensions. Rank concordance and retention of leading proteins were compared with the primary ranking. The degree-adjustment model and annotation definitions are provided in S1 Text.

### Virtual knockout analysis

Model-based virtual knockout was performed using directed, signed OmniPath relationships [21] and local CARNIVAL networks [27] reconstructed around shared OA-OP measurements. Candidate removal was represented by deletion of its incident prior-knowledge relationships and corresponding measurement constraint. The primary configuration was defined as the first evaluable baseline-knockout pair in the prespecified configuration sequence. Model-derived CARNIVAL scores summarized changes in inflammatory, extracellular-matrix, osteoclast, osteoblast, and network-state measures, with a score of at least 0.1 classified as exceeding the prespecified model-derived threshold.

Configuration sensitivity was evaluated across six combinations of measurement-set size and connector limit. Candidate evaluability, model-derived scores, above-threshold classifications, and rank concordance were compared across configurations. Network-construction parameters, solver settings, biological output panels, score calculations, and configuration definitions are provided in S1 Text and S2 Table.

### Single-cell contextual analysis, external quality control, and scTenifoldKnk analysis

Single-cell contextual analysis evaluated a prespecified candidate panel using scTenifoldKnk [42] in OA fibroblasts, OA macrophages, OP bone marrow mesenchymal stromal cells, and OP osteoclast precursors. Candidate expression and perturbation scores were calibrated against control genes matched by expression and wild-type network connectivity, with false-discovery-rate correction across evaluable candidate-compartment pairs.

An external quality-control sensitivity analysis excluded low-complexity cells or nuclei, cells with excessive mitochondrial transcript fractions, and predicted doublets [43] before recalculating candidate-compartment evidence and the cell-context component of the protein ranking. Complete cell-filtering thresholds, network-inference parameters, matched-null procedures, and sample-level retention counts are provided in S1 Text and S8 Table.

### Statistical analysis and reproducibility

All analyses were performed in R. Analytical thresholds, fallback rules, evidence-component definitions, protein-ranking weights, CARNIVAL settings, single-cell perturbation parameters, and software versions are detailed in S1 Text and S2 Table. The fully anonymized analysis code, including the primary analysis scripts and independent sensitivity-analysis scripts, is provided in S1 File. The reproducibility archive includes ranked disease signatures, shared-axis candidates, criteria-positive module pairs, pair-level empirical P values and FDR estimates, global and block-pair null summaries, protein-level evidence tables, virtual-knockout outputs, primary and quality-controlled single-cell perturbation results, cell-retention statistics, and protein-ranking sensitivity results. The main figures display selected genes and biological processes, whereas the complete ranked results and accompanying derived data are provided in S2 File.

The fully anonymized analysis code and non-identifying derived data are available from Figshare at https://doi.org/10.6084/m9.figshare.33320325. The accompanying derived data include the ranked disease signatures, shared-axis candidates, module-pair calibration results, protein-level evidence tables, virtual-knockout outputs, single-cell perturbation results, and ranking-sensitivity analyses.

## Supporting information

S1 Table Leave-one-dataset-out sensitivity analysis excluding GSE35958. (XLSX)

S2 Table Analytical parameters, evidence definitions, weighting scheme, and software versions. (XLSX)

S3 Table Permutation-based calibration of cross-disease module matching. (XLSX)

S4 Table Configuration-level stability of model-derived local CARNIVAL virtual-knockout results. (XLSX)

S5 Table Matched-null calibration of single-cell scTenifoldKnk perturbation scores. (XLSX)

S6 Table Bulk cell-composition and transcriptional-program sensitivity analyses of prioritized proteins. (XLSX)

S7 Table Hubness and annotation-density sensitivity of protein prioritization. (XLSX)

S8 Table External quality-control sensitivity of single-cell perturbation results. (XLSX)

S1 Text Supplementary methods: detailed analytical parameters and decision rules. (DOCX)

S1 Fig Leave-one-dataset-out sensitivity analysis excluding GSE35958. (A) Rank concordance between the primary OP robust rank aggregation and the analysis excluding GSE35958. (B) Rank concordance for the shared-axis candidates. (C) Stability of the primary top-10 protein ranking; crosses indicate proteins that were not retained because cross-disease direction consistency was lost. (D) Retention of primary top-ranked features at different ranking depths. For the conditional protein ranking, shared-disease robustness was recalculated from the leave-one-dataset-out results, and the other evidence dimensions were held constant.
(TIF)

S2 Fig Permutation-based calibration of cross-disease module matching. (A) Null distribution of the total number of criteria-positive OA-OP module pairs across 10,000 block-stratified module-label permutations preserving each block-specific gene universe and exact module sizes. The orange line indicates the observed count of 234, the dashed blue line indicates the null mean of 254.83, and blue shading indicates the 95% null interval of 242–267. The upper-tail empirical P value was 0.9995. (B) Observed and null-calibrated counts for the nine OA-OP block combinations. Blue points and horizontal intervals indicate null means and 95% intervals; orange points indicate observed counts. Numbers indicate pair-specific correspondences with empirical FDR < 0.05. (C) Pair-specific empirical FDR across all 464 tested module combinations. Cells are colored by pair-level -log10(empirical FDR) for the 234 criteria-positive pairs; white cells did not meet the deterministic criteria. Black dots indicate the 53 pairs with empirical FDR < 0.05. OA, osteoarthritis; OP, osteoporosis; FDR, false discovery rate.
(TIF)

S3 Fig Configuration sensitivity of local CARNIVAL virtual knockout. (A) Model-derived CARNIVAL scores for 20 candidate proteins across six combinations of measurement-set size and connector limit. Gray cells marked “NE” indicate that no evaluable local network was obtained; “KO<min” indicates that the knockout network contained fewer than the required number of measurement inputs. Bold values met the rescue-associated threshold of 0.1. (B) Configuration-level rescue profiles of HLA-DRB1, HSP90AA1, and CTSK. Crosses indicate non-evaluable configurations. (C) Numbers of attempted, evaluable, and rescue-associated configurations for the top 10 proteins. Non-evaluable configurations are reported separately from evaluable zero scores.
(TIF)

S4 Fig Matched-null calibration of single-cell perturbation scores. (A) Null-calibrated Z scores across 39 evaluable candidate-compartment pairs. Each observed score was compared with 200 control genes matched on mean log-normalized expression and wild-type weighted out-degree within the corresponding 600- or 601-gene network stratum. Asterisks indicate global FDR < 0.05, and NE indicates that no evaluable result was obtained. (B) Observed perturbation scores and corresponding matched-null means and 2.5th–97.5th percentile intervals. (C) Null-calibrated Z scores and one-sided empirical P values. Triangles indicate pairs with global FDR < 0.05. Restriction to the 100 closest controls retained the same nine globally FDR-supported pairs.
(TIF)

S5 Fig Bulk cell-composition and transcriptional-program sensitivity analysis of prioritized proteins. (A) Standardized case–control differences in MCP-counter abundance scores across four eligible mixed-tissue cohorts. (B) Ratios of composition-adjusted to unadjusted candidate effects; crosses indicate reversal of effect direction, and dots indicate adjusted effects with global FDR < 0.05. (C) Numbers of residual candidate–population associations reaching global FDR < 0.05 after removal of the case–control group effect. (D) Ratios of transcriptional-program-adjusted to unadjusted effects for HLA-DRA, HLA-DRB1, RPL7, and HSP90AA1. Crosses indicate direction reversal, and dots indicate adjusted effects with global FDR < 0.05.
(TIF)

S6 Fig Hubness and annotation-density sensitivity of protein prioritization. (A) Primary integrated score versus STRING degree. (B) Primary integrated score versus generic UniProt/HPA annotation count among the externally evaluated top 200 proteins. (C) Concordance between the primary and degree-corrected rankings. (D) Leading-protein ranks after degree correction and omission of selected evidence dimensions.
(TIF)

S7 Fig External quality-control sensitivity analysis of single-cell perturbation results. (A) Dataset-level cell counts retained and excluded by external quality control. (B) Sample-level proportions of retained cells. (C) Comparison of matched-null Z scores for the 38 candidate-compartment pairs evaluable in both the primary and quality-controlled analyses. (D) Numbers of FDR-supported compartments per candidate before and after external quality control. QC, quality control; FDR, false discovery rate.
(TIF)

S1 File Analytical code and reproducibility resources. Archive containing the primary analysis scripts, revision-analysis scripts, scoring weights, software-version records, session information, and file manifest.
(ZIP)

S2 File Processed data underlying the primary analyses. Minimal dataset containing the derived data underlying the main findings, including ranked disease signatures, shared-axis candidates, module-pair calibration results, protein-level evidence tables, virtual-knockout outputs, single-cell perturbation results, and ranking-sensitivity analyses.
(ZIP)
