# Supplementary material for: Disease-first public-data integration with local virtual knockout prioritizes shared proteins linking osteoarthritis and osteoporosis
Source: PLoS One. 2026 Oct 1;21(10):e0359583. doi: 10.1371/journal.pone.0359583 (PMC13630263; doi:10.1371/journal.pone.0359583)
Supplement: Supplementary file 1 [file pone.0359583.s009.docx]

**Supplementary Methods S1**

*Detailed analytical parameters and decision rules*

*Scope. This supplement reports the analytical parameters, thresholds, fallback rules, and score definitions supporting the methods summarized in the main manuscript.*

# 1. Bulk differential analysis and robust rank aggregation

Each bulk transcriptomic cohort was analyzed independently using limma with empirical Bayes moderation. Expression matrices were aligned to manually curated phenotype templates by sample identifier before model fitting. Platform annotations were used to map probes to genes. Features without a valid gene symbol were excluded. When several probes mapped to the same gene, the probe with the smallest adjusted P value was retained; absolute effect magnitude was used as the secondary criterion in the event of a tie.

No gene-level significance threshold was imposed before disease-level rank aggregation. Within each cohort, all successfully mapped genes were ordered primarily by adjusted P value and secondarily by absolute effect magnitude. Robust rank aggregation was conducted separately for OA and OP using the complete cohort-specific ranked lists.

Leave-one-dataset-out sensitivity analysis repeated the OP rank aggregation after exclusion of GSE35958, which comprised five OP and four control samples. Rank stability was quantified using Spearman correlation and retention within the top 20, top 100, and top 500 features. For the conditional protein-level comparison, only the shared-disease robustness dimension was recalculated; the other five evidence dimensions were retained from the primary analysis.

# 2. Gene Ontology enrichment and shared-axis construction

Gene Ontology biological-process enrichment was performed separately for the osteoarthritis and osteoporosis rank-aggregation outputs after conversion of gene symbols to Entrez identifiers. Enriched terms were corrected for multiple testing using the Benjamini-Hochberg procedure. Terms with an adjusted P value below 0.05 were retained for disease-level pathway analysis. Shared pathways required agreement in both the Gene Ontology identifier and term description between the two diseases.

For each gene, a disease-level effect was calculated as the arithmetic mean of the available cohort-specific effects within osteoarthritis or osteoporosis. Layer 1 required the mean effects for the two diseases to have the same sign. For directionally concordant genes, the direction score was defined as the smaller absolute value of the two disease-level mean effects; directionally discordant genes were excluded. Layer 2 required membership in the gene set represented by pathways significantly enriched in both diseases. The shared-axis score combined the direction score, the numbers of osteoarthritis and osteoporosis cohorts showing adjusted significance, one unit of shared-pathway support, and the number of matched module pairs containing the gene.

# 3. WGCNA construction and cross-disease module matching

Weighted gene co-expression network analysis was performed within disease-specific blocks that preserved tissue and platform coherence. A block required at least 12 samples, at least 500 genes shared across its contributing datasets, and at least 500 genes with non-zero variability. Blocks that did not satisfy these requirements were excluded. Consequently, the nine-sample osteoporosis osteogenic stromal block was not used for module construction. When several cohorts contributed to one block, cohort-specific batch effects were removed while preserving the case-control design.

The 2,000 genes with the greatest median absolute deviation were entered into network construction after removal of unsuitable samples and genes. Soft-thresholding powers from 1 to 10 were evaluated, with a fallback power of 6 when a stable estimate was unavailable. Networks used an unsigned topological overlap matrix, a minimum module size of 30, a module-merge height of 0.25, no reassignment threshold, numerical module labels, and no topological-overlap export. Module eigengenes were correlated with binary disease status. Modules were retained when the association P value was below 0.1 and the absolute correlation was at least 0.3; if no module in a block met both criteria, the two modules with the strongest trait associations were retained.

Module-level biological-process enrichment terms with an adjusted P value below 0.1 were used for functional comparison. An osteoarthritis module and an osteoporosis module were considered matched when they contained at least three overlapping genes belonging to the shared-pathway gene set, at least three overlapping enriched terms, or at least ten overlapping genes with a Jaccard index of at least 0.02. Genes occurring in retained module pairs received Layer 3 support, quantified as the number of matched pairs containing that gene.

# 4. Protein-level evidence integration and weighted prioritization

Shared-axis candidates with a positive direction score were mapped to proteins and integrated with interaction, genetic, cellular, and protein-annotation evidence. High-confidence human interactions from STRING version 12 were retained at a confidence threshold of 700, and network centrality was represented by betweenness centrality. Directed and signed signaling relationships were obtained from OmniPath. Direct GWAS Catalog and DisGeNET evaluation was restricted to the first 200 proteins after protein-layer integration.

Six evidence dimensions were defined before final ranking. Shared-disease robustness combined the cross-disease direction score, the numbers of osteoarthritis and osteoporosis cohorts showing adjusted significance, and the number of matched module pairs. Network centrality represented protein betweenness in the high-confidence interaction network. Virtual-knockout support represented predicted reversal of the shared disease network. Cell-type specificity gave equal contributions to normalized maximum candidate expression and normalized maximum perturbation score across the evaluated single-cell compartments. Genetic evidence averaged the available Open Targets disease evidence, Open Targets genetic evidence, a GWAS Catalog score capped after two direct hits, and DisGeNET evidence. Protein feasibility averaged available evidence for extracellular localization, membrane localization, Human Protein Atlas feasibility, and bone-related support.

Relative weights were specified a priori because no labeled outcome was available for empirical weight estimation. Shared-disease robustness and virtual-knockout support received weights of 0.25 each; network centrality and genetic evidence received weights of 0.15 each; and cell-type specificity and protein feasibility received weights of 0.10 each. Before integration, each dimension was independently transformed to a 0-1 scale by min-max normalization across the candidate-protein set. A dimension without finite variation contributed zero after normalization. The final priority score was calculated as the weighted sum of the six normalized dimensions.

# 5. Local CARNIVAL network reconstruction and virtual knockout

Local virtual-knockout analysis used directed, signed OmniPath relationships. Four biological output panels represented inflammation, extracellular-matrix remodeling, osteoclast activity, and osteoblast activity. The desired direction was a decrease for the first three panels and an increase for the osteoblast panel. The complete gene composition of each panel is provided in Supplementary Table S2.

For shared candidates, the osteoarthritis and osteoporosis mean effects were averaged and converted to positive or negative activity constraints. Candidate measurements were prioritized by shared-axis strength and matched-module support. The default evaluation pool contained the first 20 ranked proteins. Local networks were explored using measurement-set sizes of 8, 6, and 4 and connector limits of 3 and 2. Connector nodes required contact with at least two seed nodes and were ranked by seed contact followed by degree. A local network required at least two measured nodes and at least 15 directed, signed prior-knowledge edges before evaluation. Configurations were examined in descending order of measurement-set size; the first evaluable baseline–knockout pair defined the primary configuration.

CARNIVAL version 2.7.2 was run using lpSolve version 5.6.23 with the default linear-programming settings. The beta regularization weight was 0.2, and solver time-limit and optimality-gap parameters followed the lpSolve defaults. Candidate knockout was represented by removing all directed relationships incident to the candidate and, when present, the corresponding measurement constraint.

The model-derived CARNIVAL score combined changes in the inflammation, extracellular-matrix, osteoclast, and osteoblast output panels with reductions in abnormal network distance, disease-associated signal, overall network activity, and active-node burden. Disease-signal and network-activity reductions were divided by 100 before aggregation, and active-node reduction entered with a multiplier of 0.25. A score of at least 0.1 was classified as exceeding the prespecified model-derived threshold. Non-evaluable configurations were reported separately and were not assigned a score of zero.

Configuration sensitivity was evaluated for the 20 candidate proteins across all six combinations of measurement-set size and connector limit. Network inputs, disease measurements, biological output panels, precheck criteria, knockout definition, and model-derived score calculation were fixed across configurations. Pairwise Spearman rank correlation was calculated when both configurations contained sufficient score variation.

# 6. Single-cell contextual and perturbation analysis

Single-cell count matrices were harmonized by gene identity. Candidate-expression summaries used library-size normalization to 10,000 counts per cell followed by logarithmic transformation. Cell identities were assigned using marker-informed scores and dataset context. The prespecified perturbation panel comprised MMP9, HSP90AA1, RPL7, HLA-DRB1, TIMP1, CTNNB1, CTSK, FHL1, HLA-DRA, CLEC3B, PRG4, and NGFRAP1. The analysis included OA fibroblasts, OA macrophages, OP bone marrow mesenchymal stromal cells, and OP osteoclast precursors. Of the 48 possible candidate-compartment combinations, 39 produced evaluable perturbation scores.

Perturbation analysis required at least 80 cells in a compartment. A maximum of 250 cells was sampled using a fixed random seed. Genes detected in at least 10 cells were retained, and the 600 genes with the greatest variability were combined with the candidate gene for network inference. Three networks were generated using no more than 200 cells per network, two network components, a two-dimensional manifold, a maximum of 200 iterations, and one processing core. These thresholds governed compartment analyzability and network construction in the primary contextual analysis.

Affected genes were ordered by adjusted P value and then by absolute standardized effect. The raw perturbation score was calculated as the mean absolute standardized effect among the first 50 affected genes, and the number of affected genes with an adjusted P value below 0.05 was recorded. The raw score summarized the magnitude of transcriptomic disruption following virtual knockout. Matched-null calibration compared each observed score with scores obtained from expression- and network-degree-matched control genes.

# 7. Matched-null calibration of single-cell perturbation scores

Matched-null calibration was performed for the 39 evaluable candidate-compartment pairs. Within each compartment, mean log-normalized expression and weighted out-degree in the wild-type reference network were calculated for candidate and potential control genes. Candidate genes were excluded from the control pool. Controls were selected from the corresponding network-size stratum, distinguishing genes present among the 600 variable genes from genes introduced as the additional candidate gene. Within each stratum, matching used standardized distance in mean expression and log-transformed weighted out-degree, and the 200 closest eligible control genes were retained for each candidate-compartment pair.

Each control gene was evaluated using the same cell subsample, network-inference settings, manifold-alignment parameters, affected-gene ordering, and top-50 score definition used for the corresponding candidate. The null-calibrated Z score was calculated by subtracting the matched-control mean from the observed score and dividing by the matched-control standard deviation. A one-sided empirical P value was calculated as one plus the number of control scores at least as large as the observed score, divided by 201. Benjamini-Hochberg correction was applied across all 39 evaluable pairs. A sensitivity analysis using only the 100 closest controls retained the same nine globally FDR-supported pairs and gave concordant significance classifications for all 39 pairs. Median matching distance was summarized for each candidate-compartment pair to characterize control-match quality.

# 8. External quality-control sensitivity analysis of single-cell data

External quality control was applied separately within each public single-cell dataset before normalization and network reconstruction. Cells or nuclei with fewer than 200 detected genes were removed. Cells with mitochondrial transcript fractions above 20% in single-cell RNA-sequencing datasets or above 5% in single-nucleus RNA-sequencing datasets were excluded. Doublets were identified separately within each sample using scDblFinder version 1.20.2 without cluster-based preassignment and were removed before downstream analysis.

The quality-controlled matrices were analyzed using the same normalization, candidate panel, compartment definitions, cell-subsampling limits, gene-selection criteria, network-inference parameters, perturbation-score definition, and matched-null calibration used in the primary analysis. Ambient-RNA correction was not performed because unfiltered droplet matrices containing empty droplets were not consistently available. Cell counts before and after each filtering step were recorded at sample and dataset levels and are reported in Supplementary Table S8.

# 9. Permutation-based calibration of cross-disease module matching

Permutation calibration included the 234 criteria-positive module pairs identified by the deterministic matching rules. Block-level module membership, module Gene Ontology enrichment, and shared-pathway results were used to evaluate all 29 retained osteoarthritis modules and 16 retained osteoporosis modules, giving 464 tested cross-disease combinations.

For the gene-membership null, module labels were shuffled independently within each WGCNA block. This preserved the block-specific gene universe, the number of modules, and the exact observed size of every module while breaking the observed correspondence between gene identity and module assignment. Shared-pathway overlap and joint gene-overlap/Jaccard support were recalculated after each shuffle. Functional-term overlap was calibrated by term-count-matched hypergeometric randomization using the 2,145 significant Gene Ontology terms observed across the retained modules.

A total of 10,000 permutations was performed using random seed 20260811. Monte Carlo stability was assessed by repeating the complete calibration using random seed 20260812; the same 53 module pairs retained empirical FDR support in both runs. For each module pair and each of the three matching components, the empirical P value was calculated as (b + 1)/(B + 1), where B was 10,000 and b was the number of null statistics at least as large as the observed statistic. For the gene-based component, a null replicate was counted as at least as extreme only when both gene overlap and Jaccard similarity were at least as large as observed. Only a matching component for which the observed module pair satisfied the corresponding prespecified deterministic criterion was eligible to contribute to the pair-level test. Components that did not satisfy their corresponding observed criterion were assigned a value of one before within-pair combination. The smallest eligible component P value was multiplied by three, with an upper limit of one, to control the three within-pair matching components. Benjamini-Hochberg correction was then applied across all 464 module pairs. Pair-specific correspondence was considered statistically supported at empirical FDR below 0.05.

The global calibration compared the observed number of module pairs meeting any original deterministic matching criterion with the corresponding count in each permutation. Upper- and lower-tail empirical P values and the 2.5th and 97.5th percentiles of the null distribution were reported. The same summary was calculated for each osteoarthritis-osteoporosis block combination. Pair-level estimates are provided in Supplementary Table S3, and the global, block-pair, and pair-specific results are shown in Supplementary Figure S2.

# 10. Degree- and annotation-aware sensitivity analysis

Protein degree was calculated from the high-confidence STRING network used for primary prioritization. Degree-corrected centrality was derived from the residuals of a natural cubic-spline regression of log(1 + betweenness centrality) on log(1 + degree), using four degrees of freedom, followed by 0–1 rescaling.

Generic annotation count comprised UniProt subcellular-location and Human Protein Atlas protein-class records. Disease-evidence records were obtained from Open Targets, GWAS Catalog, and DisGeNET. Sensitivity rankings omitted network centrality, protein feasibility, or both genetic evidence and protein feasibility. Rank concordance and top-10, top-20, top-100, and top-200 retention were calculated relative to the primary ranking.

# 11. Software environment

Analyses were performed using R version 4.4.1. Principal packages included limma version 3.60.6, RobustRankAggreg version 1.2.1, clusterProfiler version 4.12.6, WGCNA version 1.74, STRINGdb version 2.16.4, OmnipathR version 3.12.4, CARNIVAL version 2.7.2, lpSolve version 5.6.23, MCPcounter version 1.2.0, org.Hs.eg.db version 3.19.1, scTenifoldKnk version 1.0.3, scTenifoldNet version 1.3, scDblFinder version 1.20.2, SingleCellExperiment version 1.28.1, BiocParallel version 1.38.0, and Matrix version 1.7-5. Complete session information is provided in the reproducibility archive.
