# Supplementary material for: Disease-first public-data integration with local virtual knockout prioritizes shared proteins linking osteoarthritis and osteoporosis
Source: PLoS One. 2026 Oct 1;21(10):e0359583. doi: 10.1371/journal.pone.0359583 (PMC13630263; doi:10.1371/journal.pone.0359583)
Supplement: Peer review history

# Peer review: Decision Letter 0

**DOI:** 10.1371/journal.pone.0359583.r001

**Authors:** Jung-Eun Kim

6 Jul 2026

Dear Dr. Yang,

Thank you for submitting your manuscript to PLOS ONE. After careful consideration, we feel that it has merit but does not fully meet PLOS ONE’s publication criteria as it currently stands. Therefore, we invite you to submit a revised version of the manuscript that addresses the points raised during the review process.

Please submit your revised manuscript by Sep 04 2026 11:59PM. If you will need more time than this to complete your revisions, please reply to this message or contact the journal office at plosone@plos.org. When you're ready to submit your revision, log on to https://www.editorialmanager.com/pone/ and select the 'Submissions Needing Revision' folder to locate your manuscript file.

- A letter that responds to each point raised by the academic editor and reviewer(s). You should upload this letter as a separate file labeled 'Response to Reviewers'.
- A marked-up copy of your manuscript that highlights changes made to the original version. You should upload this as a separate file labeled 'Revised Manuscript with Track Changes'.
- An unmarked version of your revised paper without tracked changes. You should upload this as a separate file labeled 'Manuscript'.

If you would like to make changes to your financial disclosure, please include your updated statement in your cover letter. Guidelines for resubmitting your figure files are available below the reviewer comments at the end of this letter.

If applicable, we recommend that you deposit your laboratory protocols in protocols.io to enhance the reproducibility of your results. Protocols.io assigns your protocol its own identifier (DOI) so that it can be cited independently in the future. For instructions see: https://journals.plos.org/plosone/s/submission-guidelines#loc-laboratory-protocols. Additionally, PLOS ONE offers an option for publishing peer-reviewed Lab Protocol articles, which describe protocols hosted on protocols.io. Read more information on sharing protocols at https://plos.org/protocols?utm_medium=editorial-email&utm_source=authorletters&utm_campaign=protocols.

As the corresponding author, your ORCID iD is verified in the submission system and will appear in the published article. PLOS supports the use of ORCID, and we encourage all coauthors to register for an ORCID iD and use it as well. Please encourage your coauthors to verify their ORCID iD within the submission system before final acceptance, as unverified ORCID iDs will not appear in the published article. *Only*  the individual author can complete the verification step; PLOS staff *cannot*  verify ORCID iDs on behalf of authors.

We look forward to receiving your revised manuscript.

Kind regards,

Jung-Eun Kim

Academic Editor

PLOS One

Journal Requirements:

When submitting your revision, we need you to address these additional requirements.

1.Please ensure that your manuscript meets PLOS ONE's style requirements, including those for file naming. The PLOS ONE style templates can be found at

https://journals.plos.org/plosone/s/file?id=wjVg/PLOSOne_formatting_sample_main_body.pdf and

https://journals.plos.org/plosone/s/file?id=ba62/PLOSOne_formatting_sample_title_authors_affiliations.pdf

2. Thank you for stating the following financial disclosure:

“This work was supported by grants from the Guangdong Provincial Second Hospital of Traditional Chinese Medicine Scientific research innovation Foundation (No. SEZYY2023B12).”

Please state what role the funders took in the study.  If the funders had no role, please state: "The funders had no role in study design, data collection and analysis, decision to publish, or preparation of the manuscript."

If this statement is not correct you must amend it as needed.

Please include this amended Role of Funder statement in your cover letter; we will change the online submission form on your behalf.

3. Thank you for stating the following in the Acknowledgments Section of your manuscript:

“The authors: Yu Li, Jiayin Pang, Zhichao Qi and Wenbin Yang declare that they have no conflict of interest. This work was supported by grants from the Guangdong Provincial Second Hospital of Traditional Chinese Medicine Scientific research innovation Foundation (No. SEZYY2023B12).”

We note that you have provided funding information that is not currently declared in your Funding Statement. However, funding information should not appear in the Acknowledgments section or other areas of your manuscript. We will only publish funding information present in the Funding Statement section of the online submission form.

Please remove any funding-related text from the manuscript and let us know how you would like to update your Funding Statement. Currently, your Funding Statement reads as follows:

“This work was supported by grants from the Guangdong Provincial Second Hospital of Traditional Chinese Medicine Scientific research innovation Foundation (No. SEZYY2023B12).”

Please include your amended statements within your cover letter; we will change the online submission form on your behalf.

4. Thank you for uploading your study's underlying data set. Unfortunately, the repository you have noted in your Data Availability statement does not qualify as an acceptable data repository according to PLOS's standards.

At this time, please upload the minimal data set necessary to replicate your study's findings to a stable, public repository (such as figshare or Dryad) and provide us with the relevant URLs, DOIs, or accession numbers that may be used to access these data. For a list of recommended repositories and additional information on PLOS standards for data deposition, please see https://journals.plos.org/plosone/s/recommended-repositories.

5. We note that there is identifying data in the Supporting Information file “plos_one_code.zip” Due to the inclusion of these potentially identifying data, we have removed this file from your file inventory. Prior to sharing human research participant data, authors should consult with an ethics committee to ensure data are shared in accordance with participant consent and all applicable local laws.

Data sharing should never compromise participant privacy. It is therefore not appropriate to publicly share personally identifiable data on human research participants. The following are examples of data that should not be shared:

-Name, initials, physical address

-Ages more specific than whole numbers

-Internet protocol (IP) address

-Specific dates (birth dates, death dates, examination dates, etc.)

-Contact information such as phone number or email address

-Location data

-ID numbers that seem specific (long numbers, include initials, titled “Hospital ID”) rather than random (small numbers in numerical order)

Data that are not directly identifying may also be inappropriate to share, as in combination they can become identifying. For example, data collected from a small group of participants, vulnerable populations, or private groups should not be shared if they involve indirect identifiers (such as sex, ethnicity, location, etc.) that may risk the identification of study participants.

Additional guidance on preparing raw data for publication can be found in our Data Policy (https://journals.plos.org/plosone/s/data-availability#loc-human-research-participant-data-and-other-sensitive-data) and in the following article: http://www.bmj.com/content/340/bmj.c181.long.

Please remove or anonymize all personal information (<specific identifying information in file to be removed>), ensure that the data shared are in accordance with participant consent, and re-upload a fully anonymized data set. Please note that spreadsheet columns with personal information must be removed and not hidden as all hidden columns will appear in the published file.

6. Please upload a new copy of Figure 1, 2, 3, 4, 5 and 6 as the detail is not clear. Please follow the link for more information:  https://journals.plos.org/plosone/s/figures.

7. Please include captions for your Supporting Information files at the end of your manuscript, and update any in-text citations to match accordingly. Please see our Supporting Information guidelines for more information: http://journals.plos.org/plosone/s/supporting-information.

8. If the reviewer comments include a recommendation to cite specific previously published works, please review and evaluate these publications to determine whether they are relevant and should be cited. There is no requirement to cite these works unless the editor has indicated otherwise.

[Note: HTML markup is below. Please do not edit.]

Reviewer's Responses to Questions

**Comments to the Author**

1. Is the manuscript technically sound, and do the data support the conclusions?

Reviewer #1: Yes

Reviewer #2: Yes

**********

2. Has the statistical analysis been performed appropriately and rigorously?

Reviewer #1: Yes

Reviewer #2: No

**********

3. Have the authors made all data underlying the findings in their manuscript fully available?

The PLOS Data policy

Reviewer #1: Yes

Reviewer #2: Yes

**********

4. Is the manuscript presented in an intelligible fashion and written in standard English?

Reviewer #1: Yes

Reviewer #2: Yes

**********

Reviewer #1: This is an interesting scientific manuscript and was read with pleasure. This omics study investigates shared between Osteoarthritis and Osteoporosis.

Strengths

- The topic is highly relevant

- Novelty; prior literature on the topic is sparse and the concept is a well thought question

- Potential to promote to academia beyond clinical care; this will help understand pathophysiology better acknowleding that the two disorders frequently overlap

- The approach of using "disease first", initial independent analysis of disorders and then later assessing the shared ones sounds good

- "Disease-first strategy" is intellectual choice

- Methodology of bioinformatics and the analysis are robust and apprpriate

- Transparent reporting - acknowldment in discussion that findings are hyothesis-generating than mechanistic. The careful text to not include causal terms is appreciated. The findings are approrpiately described rather than overclaims that cannot be claimed with the data

- Use of multiple independent datasets used - adds heterogeneity

- Providing datasets, codes and transparency in reporting - all these combined provide high credibility of the results

- Multiomic integration is appreciative and comprehensive data

- The identified pathways aligns with literature present

Comments -

My major comments or concerns are already acknowledged in the discussion by the authors as limitations. They have consistently avoided causal language - this is an excellent paper and well written.

Minor comments-

1. The small datasets might act as outliers and introduce random noise. OP stromal dataset is only 9 patients. Would consider dropping - the results from these may be spurious

2. How were weights assigned or computed, and at which stage was weighting done?

3. If authors can add more granular methodological details like thrsholds, parameters, protein ranking weights and CARNIVAL settings. This is not a concern of the study. This study is worthy of future work that several scientists might conduct, sharing details in supplement will help in reproducibilty of this work. This is because frequently validation studies' results gets diluted based on if methods were exactly the same as the original paper.

Reviewer #2: This manuscript presents an integrative, multi-layer computational framework to identify a shared molecular axis linking osteoarthritis (OA) and osteoporosis (OP). The work combines bulk transcriptomic meta-analysis (RRA signatures), WGCNA module matching, network-based protein prioritization, CARNIVAL-based virtual knockout, and single-cell perturbation analysis (scTenifoldKnk). The breadth of the integration is genuinely commendable, and the accompanying minimal dataset and code archive substantially improve transparency and reproducibility. The pipeline is logically organized, the processed outputs are traceable, and the analytical intent is clearly articulated.

My assessment is that this is a valuable hypothesis-generating resource. However, in line with the PLOS ONE criterion that conclusions must be supported by the data and that the analysis must be performed to a high technical standard, several core inferences currently rest on deterministic or permissive criteria that lack statistical calibration. I therefore recommend major revision. None of the points below question the value of the underlying idea, rather, they aim to strengthen the rigor so the conclusions can be stated with appropriate confidence. The concerns are addressable without new data generation.

Major Points

1. Statistical null models for cross-disease module matching

The identification of 234 matched module pairs (matched_module_pairs.tsv) is, as implemented, a deterministic procedure based on overlap and Jaccard thresholds. While the criteria are internally consistent, the manuscript does not establish that the observed degree of cross-disease module overlap exceeds what would be expected by chance given the module-size distributions and the shared background gene universe.

My suggestion is to introduce a permutation-based null model (e.g., label shuffling or degree/size-preserving randomization of module membership) to assign empirical significance and an FDR to the matched pairs. This would convert a descriptive overlap into a statistically supported claim and would materially strengthen the central "shared axis" narrative.

2. CARNIVAL virtual-knockout transparency and sensitivity

The virtual knockout is run in local_m8_c3 mode, and the rescue signal is highly localized, being active for only 2 of the 10 top proteins (HLA-DRB1 and HSP90AA1). Two issues arise. First, the solver configuration (ILP solver used, optimality gap, time limits, regularization) is not disclosed, which limits independent reproduction since CARNIVAL results can depend on solver and parameterization. Second, basing the rescue interpretation on a single subnetwork without sensitivity analysis leaves the result vulnerable to instability.

What can be done for adressing these points are: (a) Report the full solver configuration and version. (b) Provide a sensitivity/stability analysis, for example by varying the input subnetwork, perturbing edge weights, or repeating across alternative module selections, and report how consistently the rescue signal for HLA-DRB1 and HSP90AA1 is recovered.

3. Calibration of single-cell perturbation scores

The scTenifoldKnk perturbation scores (range ≈ 1.5–2.8 in single_cell_knockout_results.tsv / Table3) are defined in R/06_single_cell_validation.R as the mean absolute Z over the top-ranked affected genes, mean(abs(dr$Z[seq_len(top_n)])). This is a magnitude-of-disruption metric without a calibrated null distribution, so the absolute values are difficult to interpret as biologically meaningful effect sizes, and there is no threshold separating signal from background.

You can provide a null distribution for the perturbation score, for instance by knocking out matched random/control genes (matched on expression level and network degree) and reporting an empirical p-value or z-score for each candidate. This directly addresses whether the prioritized genes are perturbation outliers.

4. Cell-composition and abundance confounding

Several high-ranking proteins recur across compartments and analyses (notably HLA-DRA/DRB1, RPL7, HSP90AA1). MHC class II genes and ribosomal proteins are classic markers of cell-type composition shifts and global transcriptional/translational activity rather than disease-specific regulators. Without explicit control, the prioritized axis risks reflecting composition or abundance artifacts.

Here is my recommendation: (a) Examine and report whether candidate signals track cell-type proportion changes between conditions; consider a composition-aware or deconvolution-based check on the bulk layer. (b) Discuss, and where possible mitigate, the recurrent appearance of MHC-II and ribosomal genes as a potential confounder rather than a finding.

5. Hubness and annotation bias in prioritization

The network prioritization appears to reward high-degree nodes (e.g., HSP90AA1) and well-annotated genes. Degree centrality and annotation density are known to bias such rankings toward generic hubs independent of disease relevance.

It might be beneficial to report a degree-aware control, such as comparing candidate ranks against a degree-matched background or applying a degree-corrected centrality, and quantify the correlation between final priority scores and node degree / annotation count. If the ranking survives degree correction, this would considerably strengthen the specificity claim.

Single-Cell Quality Control

Code inspection of R/06_single_cell_validation.R confirms QC thresholds of ≥80 cells per group and ≥200 genes, with library-size normalization to 1e4, but the scTenifoldKnk step runs with qc = FALSE, and there is no mitochondrial-content or doublet filtering. Given that ambient RNA and doublets can inflate exactly the kind of housekeeping/ribosomal and MHC signals observed here, this is worth addressing.

Please add standard mitochondrial-fraction and doublet filtering (or justify their omission), and report cell counts before and after QC per dataset.

Minor Points and Reproducibility

- The minimal dataset and code archive are a real strength. To complete reproducibility, please specify exact package versions (a sessionInfo() or lockfile), since WGCNA, CARNIVAL, and scTenifoldKnk outputs are version-sensitive. This is already partly captured in R/00_install_packages.R; pinning versions would close the gap.

- Please clarify in the Methods that the workflow is a reanalysis of public data (the GEO series in source_datasets.tsv / Table1) rather than raw-data generation, so readers correctly interpret the scope of the validation.

- A brief statement of the directionality logic in shared_direction_consistent_genes.tsv (how concordant up/down direction is defined across heterogeneous platforms) would help readers assess the shared-direction claim.

- Consider softening causal language ("regulatory mechanism", "rescue") to reflect that the evidence is associative and model-based until the null-model and sensitivity analyses above are in place.

This is a thoughtfully constructed integrative study with a transparent codebase and a clear biological motivation. I look forward to a revised version and am confident the authors can address these points with the data and infrastructure they already have in hand.

**********

what does this mean?). If published, this will include your full peer review and any attached files.

If you choose “no”, your identity will remain anonymous but your review may still be made public.

**Do you want your identity to be public for this peer review?**  For information about this choice, including consent withdrawal, please see our Privacy Policy

Reviewer #1: No

Reviewer #2: **Yes:**  Nika Abdollahi

**********

[NOTE: If reviewer comments were submitted as an attachment file, they will be attached to this email and accessible via the submission site. Please log into your account, locate the manuscript record, and check for the action link "View Attachments". If this link does not appear, there are no attachment files.]

To ensure your figures meet our technical requirements, please review our figure guidelines: https://journals.plos.org/plosone/s/figures

You may also use PLOS’s free figure tool, NAAS, to help you prepare publication quality figures: https://journals.plos.org/plosone/s/figures#loc-tools-for-figure-preparation.

NAAS will assess whether your figures meet our technical requirements by comparing each figure against our figure specifications.

# Author comment: Author response to Decision Letter 1

**DOI:** 10.1371/journal.pone.0359583.r002

26 Aug 2026

1. PLOS style and file naming

Response: We have revised the submission materials to follow the PLOS ONE file-naming and supporting-information conventions. The existing title page, author list, affiliations, and corresponding-author information have been retained unchanged.

Changes made: Main figures are designated Fig1.tif–Fig6.tif. Supporting files and their manuscript captions use the S1 Text, S1 File, S1–S8 Table, and S1–S7 Fig conventions.

2. Financial disclosure and Acknowledgments

Response: The funding source remains unchanged. The funders had no role in study design, data collection and analysis, decision to publish, or preparation of the manuscript. We request that the online Funding Statement be updated to include this complete wording.

Changes made: The funding sentence was removed from the manuscript Acknowledgments. The complete funding information and role-of-funder statement are provided in the cover letter and will be entered in the Financial Disclosure field.

3. Stable public repository

Response: The minimal dataset and accompanying reproducibility code have been deposited in Figshare and are available at https://doi.org/10.6084/m9.figshare.33320325. The deposited files contain the derived, non-identifying data underlying the reported results and the fully anonymized analytical code.

Changes made: The repository record contains a minimal-data archive, an anonymized code archive, README documentation, and file manifests.

4. Identifying information in the previous code archive

Response: We rebuilt the code archive using only analytical scripts, configuration files, and software-version records required for reproducibility. Names, contact details, participant-level clinical fields, dates, addresses, and institution-specific identifiers were excluded. Public GEO and GSM identifiers were retained only as identifiers of public source datasets.

Changes made: A fully anonymized replacement archive is supplied as S1 File and through the public repository. The previously removed archive has not been reused.

5. Replacement figures

Response: We replaced all six main figures with clearer, high-resolution, individually uploaded TIFF files. These revisions concern image quality, terminology, and file presentation only; no numerical result, analytical definition, protein score, ranking, or conclusion was changed.

Changes made: The previous figure files have been replaced by Fig1.tif–Fig6.tif.

6. Supporting-information captions and citations

Response: Captions for S1 Text, S1 File, S1–S8 Tables, and S1–S7 Figs are now listed at the end of the manuscript. Their in-text citations, captions, and upload file names use matching designations.

Changes made: Supporting-information names and citations were standardized throughout the manuscript.

7. Citation recommendations

Response: Neither reviewer recommended citation of a specific publication. We therefore did not add any reference solely in response to this administrative item.

Changes made: None.

Reviewer #1:

Minor comments-

1. The small datasets might act as outliers and introduce random noise. OP stromal dataset is only 9 patients. Would consider dropping - the results from these may be spurious.

Response:

We agree that the limited sample size of GSE35958 requires explicit evaluation. GSE35958 comprised nine samples, including five OP samples and four controls. It was retained in the primary within-disease robust rank aggregation because this stage was designed to integrate evidence across heterogeneous OP cohorts while limiting dependence on any single dataset. However, GSE35958 did not enter the WGCNA analysis because its sample size was below the predefined minimum of 12 samples required for module construction.

In response to the reviewer’s suggestion, we performed an independent leave-one-dataset-out sensitivity analysis in which GSE35958 was excluded and the OP robust rank aggregation was repeated using GSE56814, GSE56815, and GSE230665. The resulting OP signature was then propagated through the shared-direction, shared-pathway, and protein-prioritization steps. For the conditional protein-ranking comparison, only the shared-disease robustness component was updated, whereas the network, virtual-knockout, single-cell, genetic, and protein-feasibility components were retained at their primary-analysis values.

Exclusion of GSE35958 produced Spearman rank correlations of 0.916 for the OP robust rank aggregation, 0.895 for the shared-axis ranking, and 0.918 for the conditional final protein ranking relative to the primary analysis. All 234 matched module pairs were unchanged because GSE35958 had already been excluded from module construction. Eight of the primary top ten proteins remained within the conditional top ten. HSP90AA1 and RPL7 were not retained because their cross-disease direction consistency was not preserved after exclusion of GSE35958. These results indicate that the overall integrated prioritization was reasonably robust, while also identifying HSP90AA1 and RPL7 as relatively cohort-sensitive candidates that should be interpreted more cautiously.

Changes made:

We added a concise description of this sensitivity analysis to the subsection “Differential analysis and disease-signature construction” in the Methods, immediately after the description of within-disease robust rank aggregation.

We added the following result to “OA and OP each exhibited robust disease signatures before shared-axis construction”, immediately after the paragraph ending with “platform-specific noise or tissue-specific outliers”:

To assess the influence of the small OP stromal-cell dataset, we repeated the OP integration after excluding GSE35958. The resulting OP, shared-axis, and conditional final protein rankings remained correlated with the primary analysis (Spearman ρ = 0.916, 0.895, and 0.918, respectively). All 234 matched module pairs were retained, and eight of the primary top ten proteins remained in the conditional top ten. HSP90AA1 and RPL7 were not retained after exclusion because their cross-disease direction consistency was not preserved (Supplementary Fig. S1 and Supplementary Table S1).

In the limitations paragraph of the Discussion, we added a brief statement indicating that the overall prioritization was stable after exclusion of GSE35958 but that HSP90AA1 and RPL7 showed greater cohort sensitivity.

The GSE35958 entry in Table 1 was clarified as five OP and four control samples, with the analytical role specified as bulk differential analysis and rank aggregation only; the dataset was not used for WGCNA because the total sample size was below 12.

A new Supplementary Figure S1 and Supplementary Table S1 provide the complete sensitivity-analysis results. No corresponding statement was added to the Abstract or Conclusion to avoid overemphasizing a secondary sensitivity analysis.

2. How were weights assigned or computed, and at which stage was weighting done?

Response:

We agree that the original statement that the evidence dimensions were “rescaled and combined” did not provide sufficient methodological detail. Because no labeled clinical outcome or experimentally validated reference ranking was available, the weights were not statistically fitted, learned from the observed results, or optimized to favor particular proteins. Instead, they represented a fixed rule-based evidence hierarchy defined before the final weighted integration.

Shared-disease robustness and virtual-knockout support were assigned weights of 0.25 each because they represented the principal cross-disease reproducibility and network-perturbation evidence. Network centrality and genetic evidence were assigned weights of 0.15 each as intermediate network-level and external human-disease evidence. Cell-type specificity and protein feasibility were assigned weights of 0.10 each as complementary cellular-context and follow-up-feasibility evidence.

Before integration, each of the six evidence dimensions was independently normalized to the 0–1 interval across the candidate-protein set. A dimension without finite variation contributed zero after normalization. Weighting was applied only during the final protein-prioritization step. It was not used in cohort-level differential analysis, robust rank aggregation, pathway enrichment, shared-axis construction, or module matching.

Changes made:

We replaced the original paragraph under “Protein-level evidence integration and final prioritization”, beginning with “Shared-axis candidates were mapped to proteins” and ending with “the final ranking reported in Table 2,” with an expanded description of the six evidence dimensions, their normalization, the rationale for their relative weights, and the stage at which weighting was applied.

The revised text explicitly reports the following weights:

Shared-disease robustness, 0.25; network centrality, 0.15; virtual-knockout support, 0.25; cell-type specificity, 0.10; genetic evidence, 0.15; and protein feasibility, 0.10.

The complete definitions, normalization rules, weight-assignment rationale, and weighting stage are now reported in Supplementary Methods S1 and Supplementary Table S2.

3. If authors can add more granular methodological details like thrsholds, parameters, protein ranking weights and CARNIVAL settings. This is not a concern of the study. This study is worthy of future work that several scientists might conduct, sharing details in supplement will help in reproducibilty of this work. This is because frequently validation studies' results gets diluted based on if methods were exactly the same as the original paper.

Response:

We agree and have substantially expanded methodological reporting without changing the original analytical framework. The revised Methods now specify the decision rules governing differential analysis, robust rank aggregation, pathway analysis, WGCNA, cross-disease module matching, protein-level evidence integration, local CARNIVAL reconstruction, virtual-knockout scoring, and single-cell contextual analysis.

The supplementary description now reports, among other details, the probe-to-gene collapse rule; the ordering and inclusion criteria used for robust rank aggregation; multiple-testing thresholds for biological-process enrichment; WGCNA block-eligibility criteria, soft-threshold selection, module size and merging settings, module–trait criteria, and cross-disease module-matching thresholds; the definitions and normalization of all six protein-level evidence dimensions; and the complete weighting scheme.

For CARNIVAL, we now report the software version, solver, regularization setting, candidate pool, local-network construction sequence, connector limits, minimum network requirements, four biological output panels, knockout implementation, and rescue-score composition. CARNIVAL version 2.7.2 was used with the lpSolve solver and a beta regularization weight of 0.2. No customized solver time limit or optimality-gap threshold was introduced.

For the single-cell layer, we now report the normalization procedure, marker-supported annotation rule, evaluated compartments, minimum number of cells, sampling limit and random seed, gene-detection and variability filters, network number, maximum cells per network, manifold dimensions, iteration limit, processing cores, and perturbation-score definition.

Changes made:

We expanded the corresponding main-text Methods subsections while keeping the principal manuscript concise:

• “Differential analysis and disease-signature construction”

• “Shared pathological axis and disease-associated module matching”

• “Protein-level evidence integration and final prioritization”

• “Virtual knockout analysis”

• “Single-cell validation and scTenifoldKnk analysis”

• “Statistical analysis and reproducibility”

We added Supplementary Methods S1, organized into six directly corresponding methodological sections:

1. Bulk differential analysis and robust rank aggregation

2. Gene Ontology enrichment and shared-axis construction

3. WGCNA construction and cross-disease module matching

4. Protein-level evidence integration and weighted prioritization

5. Local CARNIVAL network reconstruction and virtual knockout

6. Single-cell contextual and perturbation analysis

We also added Supplementary Table S2, which provides a structured record of the evidence-dimension definitions, weighting scheme, bulk and pathway parameters, WGCNA and module-matching criteria, CARNIVAL settings and biological output panels, single-cell settings, and exact software versions. These additions improve reproducibility and reporting transparency but do not change the primary analytical definitions or results.

Reviewer #2:

Major Points

1. Statistical null models for cross-disease module matching

The identification of 234 matched module pairs (matched_module_pairs.tsv) is, as implemented, a deterministic procedure based on overlap and Jaccard thresholds. While the criteria are internally consistent, the manuscript does not establish that the observed degree of cross-disease module overlap exceeds what would be expected by chance given the module-size distributions and the shared background gene universe.

My suggestion is to introduce a permutation-based null model (e.g., label shuffling or degree/size-preserving randomization of module membership) to assign empirical significance and an FDR to the matched pairs. This would convert a descriptive overlap into a statistically supported claim and would materially strengthen the central "shared axis" narrative.

Response

We thank the reviewer for this important and constructive comment. We agree that the original deterministic matching criteria, although prespecified and internally consistent, were not sufficient to establish whether the observed cross-disease module correspondence exceeded that expected by chance after accounting for module-size distributions and the shared gene background.

In the original analysis, module matching was designed as a structured Layer 3 evidence component rather than as a formal statistical enrichment test. The 234 module pairs were retained when they satisfied at least one prespecified overlap criterion involving shared-pathway genes, enriched functional terms, or joint gene overlap and Jaccard similarity. This approach allowed module-level evidence to contribute to the multilayer protein-prioritization framework while preserving the independently constructed OA and OP network structures. However, we recognize that referring to these pairs simply as “matched module pairs” could be interpreted as implying that all 234 pairs represented statistically significant cross-disease correspondence. The original manuscript did not distinguish sufficiently between deterministic criteria-positive pairs and statistically calibrated pair-specific correspondence. We therefore accepted the reviewer’s recommendation.

We added an independent permutation-based null analysis without changing the original WGCNA modules, deterministic matching thresholds, Layer 3 definitions, candidate pool, scoring weights, or protein ranking. The original 234 criteria-positive pairs were first reconstructed exactly from the frozen primary outputs. All 29 retained OA modules and 16 retained OP modules were then evaluated, corresponding to 464 possible OA-OP module combinations.

For the gene-membership null, module labels were shuffled independently within each WGCNA block over 10,000 permutations. This procedure preserved the block-specific gene universe, the number of modules, and the exact observed size of every module while disrupting the association between gene identity and module assignment. Shared-pathway overlap and joint gene-overlap/Jaccard support were recalculated after each permutation. Functional-term overlap was calibrated using term-count-matched randomization over the 2,145 significant Gene Ontology terms observed across the retained modules.

Empirical P values were calculated separately for the three original matching components. Only components for which the observed module pair satisfied the corresponding prespecified matching criterion were eligible to contribute to pair-level significance. The smallest eligible component P value was Bonferroni-adjusted across the three matching components, followed by Benjamini-Hochberg correction across all 464 tested module pairs.

Of the 234 pairs meeting the original deterministic criteria, 53 retained pair-specific support at empirical FDR < 0.05. An independent repeat using a second random seed recovered the same 53 pairs, indicating stable identification of the statistically supported subset. However, the total number of criteria-positive pairs did not exceed the global null expectation: 234 pairs were observed compared with a null mean of 254.83 and a 95% null interval of 242-267; the upper-tail empirical P value was 0.9995.

We therefore revised the interpretation of the module layer. The 234 pairs are now described as “criteria-positive module pairs,” whereas the 53 pairs meeting empirical FDR < 0.05 are described as “statistically supported pair-specific correspondences.” We no longer interpret the total number of 234 pairs as evidence of globally enriched OA-OP module similarity. Instead, the revised manuscript states that statistical module-level support is concentrated in selected cross-disease module pairs.

We retained the original protein scores and ranking because the permutation analysis was designed to calibrate the inferential strength of the module correspondences, not to redefine the prespecified primary scoring framework after observing the results. Retrospectively changing the candidate pool, weights, or score calculation on the basis of the new null results would introduce a post hoc modification to the primary analysis. The calibration was therefore added as an independent statistical annotation. This decision also reflects the multilayer nature of the ranking: module correspondence was one contributory dimension and was not the sole basis for protein prioritization.

Among the top 10 proteins, HLA-DRB1, HSP90AA1, CTSK, RPL7, CLEC3B, and SPP1 occurred in at least one FDR-supported module pair. PRG4, HLA-DRA, TIMP1, and APOE did not receive an FDR-supported module-pair annotation but retained their original ranks through complementary evidence from disease-level robustness, network context, cell-context support, genetic evidence, protein feasibility, or local virtual-knockout analysis. Consequently, the revised manuscript retains the overall multilayer protein-prioritization conclusion while narrowing the specific network-level claim from global module convergence to selected pair-specific correspondence.

This calibrated interpretation is also the reason that the revised manuscript continues to describe a shared OA-OP pathological axis. That conclusion does not depend solely on the total number of module pairs; it is supported jointly by concordant disease-level expression, shared pathway enrichment, selected statistically supported module correspondences, protein-level evidence integration, local CARNIVAL analysis, and single-cell contextualization. The revised text therefore preserves the overall hypothesis-generating framework while avoiding an unsupported claim of globally significant module similarity.

Changes Made

1. Abstract: We replaced the statement that cross-disease comparison “identified 234 matched module pairs” with a calibrated description distinguishing 234 criteria-positive pairs from the 53 pairs retaining empirical FDR < 0.05. We also clarified that the total number of criteria-positive pairs did not exceed the global null expectation.

2. Results: We rewrote the Layer 3 results to report all 464 tested OA-OP module combinations, the 234 criteria-positive pairs, the 53 FDR-supported pairs, the second-seed stability result, and the global null statistics. The revised Results now describe selected pair-specific correspondence rather than globally enriched module matching.

3. Protein-level Results: We added the FDR-supported module-pair counts for the top 10 proteins and clarified that the independent calibration did not alter the original composite scores or ranking.

4. Methods: We corrected and fully stated the three original deterministic module-matching criteria. We then added the complete permutation procedure, including preservation of block-specific gene universes and exact module sizes, functional-term randomization, empirical P-value calculation, within-pair Bonferroni correction, Benjamini-Hochberg correction across 464 pairs, and the independent second-seed stability analysis.

5. Discussion: We revised the interpretation of the module layer to state that statistical support was concentrated in selected module pairs and did not demonstrate a global excess of OA-OP module similarity.

6. Limitations: We added the absence of global module-pair enrichment as an explicit limitation and clarified that the module layer should be interpreted as pair-specific concordance.

7. Conclusion: We replaced the broad phrase “matched modules” with “selected pair-specific module correspondences.” The overall multilayer and hypothesis-generating conclusion was retained.

8. Figure 1: Panel B now includes “criteria-positive module pairs” followed by “pair-specific permutation calibration.” Panel C now identifies Layer 3 as deterministic module evidence and states that pair-level significance was calibrated independently. The original Layer 3 count of 810 was retained because the permutation analysis did not redefine the primary candidate set.

9. Figure 3: Panel A now identifies the displayed counts as cumulative evidence coverage under deterministic criteria. Panel C distinguishes 234 criteria-positive pairs from 53 FDR-supported pairs. Panel D now displays the original criteria-positive counts as light bars and the FDR-supported subset as dark overlays, together with the global null result.

10. Figure 4 and Table 2: The original protein scores were retained. Table 2 now reports the number of FDR-supported module pairs for each leading protein, and the figure/table notes clarify that permutation calibration was not used to reweight or rerank candidates.

11. Supplementary Figure S2: We added the global permutation distribution, block-pair calibration, and pair-specific empirical FDR matrix across all 464 module combinations.

12. Supplementary Table S3: We added the global null summary, block-pair results, empirical P values and FDR estimates for the 234 criteria-positive pairs, and the mapping between FDR-supported module pairs and the leading proteins.

13. Supplementary Methods: We added a dedicated section describing the null-model construction, preserved quantities, empirical significance calculation, multiple-testing correction, and stability assessment.

14. Reproducibility statement: We expanded the listed reproducible outputs to include criteria-positive module pairs, pair-level empirical P values and FDR estimates, and global and block-pair null summaries.

2. CARNIVAL virtual-knockout transparency and sensitivity

The virtual knockout is run in local_m8_c3 mode, and the rescue signal is highly localized, being active for only 2 of the 10 top proteins (HLA-DRB1 and HSP90AA1). Two issues arise. First, the solver configuration (ILP solver used, optimality gap, time limits, regularization) is not disclosed, which limits independent reproduction since CARNIVAL results can depend on solver and parameterization. Second, basing the rescue interpretation on a single subnetwork without sensitivity analysis leaves the result vulnerable to instability.

What can be done for adressing these points are: (a) Report the full solver configuration and version. (b) Provide a sensitivity/stability analysis, for example by varying the input subnetwork, perturbing edge weights, or repeating across alternative module selections, and report how consistently the rescue signal for HLA-DRB1 and HSP90AA1 is recovered.

Response: We thank the reviewer for identifying the need to distinguish the primary local CARNIVAL result from scores that remain stable across alternative network configurations. We expanded both solver reporting and configuration-sensitivity assessment.

The primary analysis used CARNIVAL version 2.7.2 with lpSolve version 5.6.23, a beta regularization weight of 0.2, and the software-default linear-programming settings. No customized solver time limit or optimality-gap criterion was imposed.

The 20 prespecified candidates were evaluated across six combinations of measurement-set size and connector limit. Of 120 protein-configuration combinations, 62 were evaluable. HLA-DRB1 exceeded the prespecified model-derived threshold in all four evaluable configurations and retained a score of 2.25. HSP90AA1 exceeded the threshold in four of six configurations, with scores of 1.25 under the eight- and six-measurement settings and 0 under both four-measurement settings. CTSK was evaluable in all six configurations and had a score of 0 throughout. These results identify HLA-DRB1 as the more configuration-stable model-derived candidate and qualify the HSP90AA1 result as configuration-dependent.

Changes made: Complete solver settings, score definitions, evaluability criteria, and configuration-level results were added to the Methods, S1 Text, S2 Table, S3 Fig, and S4 Table. Figure 5 and Table 2 were revised to use model-derived CARNIVAL terminology. No analytical score, threshold, or protein ranking was changed.

3. Calibration of single-cell perturbation scores

The scTenifoldKnk perturbation scores (range ≈ 1.5–2.8 in single_cell_knockout_results.tsv / Table3) are defined in R/06_single_cell_validation.R as the mean absolute Z over the top-ranked affected genes, mean(abs(dr$Z[seq_len(top_n)])). This is a magnitude-of-disruption metric without a calibrated null distribution, so the absolute values are difficult to interpret as biologically meaningful effect sizes, and there is no threshold separating signal from background.

You can provide a null distribution for the perturbation score, for instance by knocking out matched random/control genes (matched on expression level and network degree) and reporting an empirical p-value or z-score for each candidate. This directly addresses whether the prioritized genes are perturbation outliers.

Response

We thank the reviewer for highlighting that the original scTenifoldKnk perturbation scores lacked a gene-matched reference distribution and could therefore be influenced by baseline expression and network connectivity. We agree that raw perturbation scores alone cannot establish whether a candidate produces an unusually strong network effect relative to genes with comparable expression and topological properties.

We therefore performed a matched-null calibration for all 39 evaluable candidate–compartment pairs. For each candidate, 200 control genes were selected from the same 600- or 601-gene network-size stratum and matched according to mean log-normalized expression and log-transformed wild-type weighted out-degree. Each matched control was evaluated using the same cell subsample, network-inference procedure, manifold-alignment settings, affected-gene ordering, and top-50 perturbation-score definition as the corresponding candidate. The observed score was then compared with its matched-null distribution. We calculated a null-calibrated Z score and a one-sided empirical P value using the plus-one correction, followed by Benjamini–Hochberg adjustment across all 39 evaluable pairs.

Eleven candidate–compartment pairs had nominal empirical P values below 0.05, and nine remained supported after global false-discovery-rate correction. These comprised CTSK, CLEC3B, TIMP1, PRG4, and HSP90AA1 in OA macrophages; RPL7 in OA fibroblasts; CLEC3B in OP bone marrow mesenchymal stromal cells; and TIMP1 and RPL7 in OP osteoclast precursors. In contrast, several candidates with relatively high raw perturbation scores, including MMP9, HLA-DRA, HLA-DRB1, and CTSK in OP compartments, did not exceed their matched-null backgrounds after correction. Thus, the calibration distinguished absolute perturbation magnitude from effects that were unusually large relative to expression- and degree-matched genes.

We also performed a sensitivity analysis restricted to the 100 closest matched controls. This analysis retained the same nine globally FDR-supported pairs and produced concordant significance classifications for all 39 evaluable pairs. Matching diagnostics were additionally examined and are now reported transparently, including the comparatively larger matching distances for OA macrophage PRG4 and HSP90AA1. These results refine the single-cell findings as calibrated, compartment-specific contextual evidence rather than direct mechanistic validation.

Changes made

1. We expanded the Single-cell contextual analysis subsection of the Methods to specify the evaluated cellular compartments, candidate panel, scTenifoldKnk settings, and definition of the raw perturbation score.

2. We added a dedicated Matched-null calibration of single-cell perturbation scores subsection to Supplementary Methods S1. This section now describes the network-size strata, matching variables, standardized matching distance, selection of 200 matched controls, null Z-score calculation, plus-one empirical P-value calculation, Benjamini–Hochberg correction across 39 tests, and nearest-100-control sensitivity analysis.

3. We rewrote the corresponding Results subsection to report the number of evaluable pairs, nominally supported pairs, globally FDR-supported pairs, compartment-specific results, matching diagnostics, and sensitivity-analysis results.

4. We revised Figure 6C–D to present matched-null Z scores and the leading calibrated OA and OP candidate–compartment pairs instead of relying only on raw perturbation scores.

5. We added Supplementary Figure S4, which reports the complete matched-null calibration, observed scores relative to matched-null intervals, empirical significance results, and the nearest-100-control sensitivity analysis.

6. We revised Table 3 to report the nine globally FDR-supported candidate–compartment pairs, including observed scores, matched-null summaries, Z scores, empirical P values, adjusted P values, and matching diagnostics.

7. We added Supplementary Table S5, containing results for all 39 evaluable pairs, nearest-100 sensitivity results, matching diagnostics, identities of all 7,800 matched controls, complete matched-control score distributions, group information, and variable definitions.

8. We revised the Discussion and limitations to distinguish raw perturbation magnitude from matched-null outlier status and to describe the single-cell findings as computational, compartment-specific contextual support requiring independent experimental confirmation.

4. Cell-composition and abundance confounding

Several high-ranking proteins recur across compartments and analyses (notably HLA-DRA/DRB1, RPL7, HSP90AA1). MHC class II genes and ribosomal proteins are classic markers of cell-type composition shifts and global transcriptional/translational activity rather than disease-specific regulators. Without explicit control, the prioritized axis risks reflecting composition or abundance artifacts.

Here is my recommendation: (a) Examine and report whether candidate signals track cell-type proportion changes between conditions; consider a composition-aware or deconvolution-based check on the bulk layer. (b) Discuss, and where possible mitigate, the recurrent appearance of MHC-II and ribosomal genes as a potential confounder rather than a finding.

Response

We agree that the recurrent prioritization of MHC-II and ribosomal proteins could partly reflect differences in cellular abundance or broader transcriptional activity rather than disease-specific regulation. We therefore performed two complementary sensitivity analyses. First, inferred immune and stromal abundance scores were examined in four eligible mixed-tissue cohorts, and candidate effects were re-estimated after adjustment for the leading abundance-score components. None of the 40 population-by-cohort differences remained significant after global FDR correction, and 36 of 40 candidate–cohort effects retained their original direction. However, 50 of 400 residual candidate–population associations remained significant, indicating that some candidates continued to track cellular abundance. Second, HLA-DRA, HLA-DRB1, RPL7, and HSP90AA1 were adjusted for matched MHC-II, ribosomal, or protein-folding transcriptional programs. Direction was retained in 24 of 27 evaluable comparisons, although the MHC-II candidates showed greater attenuation. We have therefore revised the manuscript to interpret MHC-II and ribosomal proteins as context-sensitive candidates rather than confirmed disease-specific regulators.

Change made

We added a bulk cell-composition and transcriptional-program sensitivity analysis to the Methods and Results, qualified the interpretation of HLA-DRA, HLA-DRB1, and RPL7 in the Discussion and Limitations, and added revised Supplementary Figure S5 and Supplementary Table S6 containing the complete four-cohort results.

5. Hubness and annotation bias in prioritization

The network prioritization appears to reward high-degree nodes (e.g., HSP90AA1) and well-annotated genes. Degree centrality and annotation density are known to bias such rankings toward generic hubs independent of disease relevance.

It might be beneficial to report a degree-aware control, such as comparing candidate ranks against a degree-matched background or applying a degree-corrected centrality, and quantify the correlation between final priority scores and node degree / annotation count. If the ranking survives degree correction, this would considerably strengthen the specificity claim.

Response:

We thank the reviewer for highlighting the potential influence of network hubness and annotation density on protein prioritization. We agree that betweenness centrality may favor highly connected proteins and that well-characterized proteins may receive greater support from public annotation resources. We therefore performed independent degree-aware and annotation-aware sensitivity analyses using the original candidate pool and prespecified evidence weights.

The primary integrated score was moderately correlated with STRING degree (Spearman ρ = 0.476, P < 2.2 × 10−16). After replacing the original centrality dimension with degree-corrected centrality, 9 of the primary top 10 and 165 of the top 200 proteins were retained, with a rank correlation of ρ = 0.882 among the primary top 200. Complete omission of network centrality retained 9 of the top 10 and 190 of the top 200 proteins. HLA-DRB1 and HSP90AA1 remained ranked first and second in both analyses.

Among the 200 proteins evaluated using external resources, the primary score correlated with generic UniProt/HPA annotation count (ρ = 0.388, P = 1.36 × 10−8). After omission of protein feasibility, this association decreased to ρ = 0.106 and was no longer statistically significant (P = 0.133). Simultaneous omission of genetic evidence and protein feasibility retained 7 of the primary top 10 and 185 of the top 200 proteins. HLA-DRB1 and HSP90AA1 remained first and second, whereas SPP1 and APOE moved to ranks 34 and 38, respectively. These results indicate that the two leading candidates were not prioritized solely because of network degree, while the positions of SPP1 and APOE depended more strongly on external evidence dimensions.

Change made:

We added the degree-correction procedure, annotation-density definitions, and evidence-dimension omission analyses to the Methods and Supplementary Methods S1. The corresponding correlations, rank-retention results, and candidate-specific rank changes were added to the protein-prioritization Results. The Discussion now distinguishes candidates supported across multiple evidence dimensions from those more dependent on specific external evidence sources, and the relevant limitation was expanded. Complete results are presented in Supplementary Figure S6 and Supplementary Table S7. The primary protein ranking and the values reported in Figure 4 and Table 2 were not changed.

Single-Cell Quality Control

Code inspection of R/06_single_cell_validation.R confirms QC thresholds of ≥80 cells per group and ≥200 genes, with library-size normalization to 1e4, but the scTenifoldKnk step runs with qc = FALSE, and there is no mitochondrial-content or doublet filtering. Given that ambient RNA and doublets can inflate exactly the kind of housekeeping/ribosomal and MHC signals observed here, this is worth addressing.

Please add standard mitochondrial-fraction and doublet filtering (or justify their omission), and report cell counts before and after QC per dataset.

Response

We thank the reviewer for identifying the limitations of the original single-cell quality-control procedure. We agree that filtering based only on detected-gene and compartment-size criteria did not directly address excessive mitochondrial transcript fractions or predicted doublets. We therefore performed an independent external quality-control sensitivity analysis before normalization and network reconstruction.

Cells or nuclei with fewer than 200 detected genes were excluded. Mitochondrial-transcript thresholds were set at 20% for single-cell RNA-sequencing data and 5% for single-nucleus RNA-sequencing data. Doublets were identified separately within each sample using scDblFinder version 1.20.2 and removed before downstream analysis. The quality-controlled matrices were then processed using the same normalization, compartment definitions, candidate panel, cell-subsampling limits, gene-selection criteria, scTenifoldKnk parameters, perturbation-score definition, and matched-null calibration procedure as the primary analysis.

External quality control retained 125,090 of 161,470 cells. The quality-controlled analysis identified 10 globally FDR-supported candidate-compartment pairs. Pair-level localization was sensitive to quality control: only HSP90AA1 and TIMP1 in OA macrophages remained supported in the same compartment, although five of the six genes supported in the primary analysis retained evidence in at least one quality-controlled compartment. Importantly, recalculation of the cell-context evidence retained all primary top 10 proteins and 19 of the primary top 20 proteins, with an overall rank correlation greater than 0.999; HLA-DRB1 and HSP90AA1 remained ranked first and second.

Ambient-RNA correction could not be applied consistently because unfiltered droplet matrices containing empty droplets were not available for all three public datasets. We now report this constraint explicitly. The revised interpretation therefore treats the single-cell results as quality-control-sensitive cellular contextual evidence rather than fixed cell-type localization or experimental validation.

Changes made

We added the external quality-control thresholds, sample-wise doublet detection, and quality-controlled reanalysis procedure to the Methods. We added dataset-, sample-, and compartment-level cell-retention statistics and the comparison between primary and quality-controlled perturbation results to the Results. The Discussion and Limitations now state that precise candidate-compartment assignments were not uniformly stable, whereas the overall protein-ranking hierarchy remained stable. Table 1 now specifies the role of each single-cell dataset, and Table 3 directly compares primary and quality-controlled candidate-compartment results. Figure 6 remains the primary matched-null analysis, while Supplementary Figure S7 and Supplementary Table S8 report the complete external quality-control sensitivity analysis.

Minor Points and Reproducibility

- The minimal dataset and code archive are a real strength. To complete reproducibility, please specify exact package versions (a sessionInfo() or lockfile), since WGCNA, CARNIVAL, and scTenifoldKnk outputs are version-sensitive. This is already partly captured in R/00_install_packages.R; pinning versions would close the gap.

Response

Thank you for highlighting the importance of version-specific reproducibility. We agree that WGCNA, CARNIVAL, and scTenifoldKnk outputs can depend on the software environment. We have therefore reported the exact R and package versions used for the analyses in Supplementary Methods S1 and included the complete session information and package-version manifest in the reproducibility archive. These additions document the computational environment without altering the analytical definitions, parameters, or results.

Changes made

We added a dedicated “Software environment” subsection to Supplementary Methods S1, specifying R version 4.4.1 and the exact versions of the principal packages, including WGCNA 1.74, CARNIVAL 2.7.2, scTenifoldKnk 1.0.3, limma 3.60.6, RobustRankAggreg 1.2.1, clusterProfiler 4.12.6, and the supporting single-cell and network-analysis packages. Complete sessionInfo() and package-version records were also included in the reproducibility archive.

- Please clarify in the Methods that the workflow is a reanalysis of public data (the GEO series in source_datasets.tsv / Table1) rather than raw-data generation, so readers correctly interpret the scope of the validation.

Response

We agree that the scope of the study should be explicit. The opening paragraph of the Methods now states that this study was a secondary reanalysis of publicly available, de-identified GEO transcriptomic datasets and that no new participant recruitment, biospecimen collection, or transcriptomic data generation was undertaken. The GEO series and their analytical roles are listed in Table 1.

Changes made

We revised the opening of the Methods to distinguish secondary public-data reanalysis from primary data generation and retained the complete dataset-level description in Table 1.

- A brief statement of the directionality logic in shared_direction_consistent_genes.tsv (how concordant up/down direction is defined across heterogeneous platforms) would help readers assess the shared-direction claim.

Response

Thank you for requesting clarification of the cross-platform directionality rule. We have clarified that directionality was determined from cohort-specific case-control log-fold-change estimates rather than by directly comparing expression intensities across platforms. For each mapped gene, cohort-specific effects were averaged separately within OA and OP. Positive mean effects in both diseases defined concordant upregulation, negative mean effects in both diseases defined concordant downregulation, and genes with opposing signs were excluded from the shared axis. The full direction-score definition remains available in Supplementary Methods S1.

Changes made

We added a concise definition of concordant upregulation and downregulation to the main Methods and retained the mathematical direction-score rule in Supplementary Methods S1.

- Consider softening causal language ("regulatory mechanism", "rescue") to reflect that the evidence is associative and model-based until the null-model and sensitivity analyses above are in place.

Response

We agree that the computational outputs should not be interpreted as experimental evidence of causality or biological rescue. We therefore replaced unqualified “rescue” claims with “model-derived CARNIVAL score” or “above the prespecified model-derived threshold” throughout the Abstract, Results, Discussion, Methods, tables, and figure legends. “Virtual knockout” was retained as the established name of the computational procedure, but its outputs are now consistently described as model-derived. The Limitations and Conclusion explicitly state that these analyses provide functional prioritization and do not establish causal regulators or therapeutic effects.

Changes made

We revised the manuscript-facing terminology for CARNIVAL results, removed statements implying confirmed network rescue, and updated the corresponding Table 2 categories and Figure 4, Figure 5, and Supplementary Figure S3 labels. No scores, thresholds, rankings, or analytical results were changed.

Attachment Submitted filename: Point-by-point response.docx

# Editor report: Decision Letter 1

**DOI:** 10.1371/journal.pone.0359583.r003

**Authors:** Jung-Eun Kim

15 Sep 2026

<p>Disease-first public-data integration with local virtual knockout prioritizes shared proteins linking osteoarthritis and osteoporosis

PONE-D-26-26842R1

Dear Dr. Yang,

We’re pleased to inform you that your manuscript has been judged scientifically suitable for publication and will be formally accepted for publication once it meets all outstanding technical requirements.

Within one week, you’ll receive an e-mail detailing the required amendments. When these have been addressed, you’ll receive a formal acceptance letter and your manuscript will be scheduled for publication.

An invoice will be generated when your article is formally accepted. Please note, if your institution has a publishing partnership with PLOS and your article meets the relevant criteria, all or part of your publication costs will be covered. Please make sure your user information is up-to-date by logging into Editorial Manager at Editorial Manager® and clicking the ‘Update My Information' link at the top of the page. For questions related to billing, please contact billing support.

If your institution or institutions have a press office, please notify them about your upcoming paper to help maximize its impact. If they’ll be preparing press materials, please inform our press team as soon as possible -- no later than 48 hours after receiving the formal acceptance. Your manuscript will remain under strict press embargo until 2 pm Eastern Time on the date of publication. For more information, please contact onepress@plos.org.

Kind regards,

Jung-Eun Kim

Academic Editor

PLOS One

Additional Editor Comments (optional):

Reviewers' comments:

# Editor report: Acceptance letter

**DOI:** 10.1371/journal.pone.0359583.r004

**Authors:** Jung-Eun Kim

PONE-D-26-26842R1

PLOS One

Dear Dr. Yang,

I'm pleased to inform you that your manuscript has been deemed suitable for publication in PLOS One. Congratulations! Your manuscript is now being handed over to our production team.

At this stage, our production department will prepare your paper for publication. This includes ensuring the following:

* All references, tables, and figures are properly cited

* All relevant supporting information is included in the manuscript submission,

* There are no issues that prevent the paper from being properly typeset

You will receive further instructions from the production team, including instructions on how to review your proof when it is ready. Please keep in mind that we are working through a large volume of accepted articles, so please give us a few days to review your paper and let you know the next and final steps.

Lastly, if your institution or institutions have a press office, please let them know about your upcoming paper now to help maximize its impact. If they'll be preparing press materials, please inform our press team within the next 48 hours. Your manuscript will remain under strict press embargo until 2 pm Eastern Time on the date of publication. For more information, please contact onepress@plos.org.

You will receive an invoice from PLOS for your publication fee after your manuscript has reached the completed accept phase. If you receive an email requesting payment before acceptance or for any other service, this may be a phishing scheme. Learn how to identify phishing emails and protect your accounts at https://explore.plos.org/phishing.

If we can help with anything else, please email us at customercare@plos.org.

Thank you for submitting your work to PLOS One and supporting open access.

Kind regards,

PLOS One Editorial Office Staff

on behalf of

Dr Jung-Eun Kim

Academic Editor

PLOS One
